# The level of embryonation influences detection of *Ostertagia ostertagi* eggs by semi-quantitative PCR

**DOI:** 10.1186/s13071-016-1657-4

**Published:** 2016-06-29

**Authors:** Markus Drag, Johan Höglund, Peter Nejsum, Stig M. Thamsborg, Heidi L. Enemark

**Affiliations:** Section for Parasitology and Aquatic Diseases, Department of Veterinary Disease Biology, Faculty of Health and Medical Sciences, University of Copenhagen, Frederiksberg C, Denmark; Section for Bacteriology, Pathology and Parasitology, National Veterinary Institute, Technical University of Denmark, Frederiksberg C, Denmark; Department of Biomedical Sciences and Veterinary Public Health, Section for Parasitology, Swedish University of Agricultural Sciences, Uppsala, Sweden; Norwegian Veterinary Institute, PO Box 750 Sentrum, Oslo, N-0106 Norway

**Keywords:** *Ostertagia ostertagi*, Egg development, First-stage larvae, Real-time semi-quantitative PCR, ITS2

## Abstract

**Background:**

The Internal Transcribed Spacer 2 (ITS2) is a candidate diagnostic marker of the pathogenic cattle nematode *Ostertagia ostertagi*. The aims of this study were: (i) to document and quantify how the development of *O. ostertagi* eggs affects ITS2 copies under different storage conditions, and (ii) to suggest optimal storage conditions for faecal samples in a diagnostic pipeline that involves detection and semi-quantification by real-time semi-quantitative polymerase chain reaction (qPCR).

**Findings:**

Eggs of *Ostertagia ostertagi* were obtained from fresh faeces and stored at 4 °C or 25 °C under aerobic or anaerobic (vacuum packing) conditions. Development was monitored by microscopy for up to 336 h, and the ITS2 copies were determined by qPCR from a fixed number of parasites. Under aerobic conditions at 25 °C, embryonation and a significant increase of ITS2 copies (*P* < 0.0001) were observed after 12 h. At 4 °C, embryonation occurred after 168 h with a trend towards increased ITS2 copies. Anaerobic conditions inhibited egg development at both temperatures and no significant increase in ITS2 copies was noticed (*P* = 0.90). ITS2 copies were analysed for each parasite stage: first-stage larvae (L1) exhibited significantly higher copy numbers (20,353 ± 1,950) than unembryonated eggs (568 ± 168; *P* < 0.0001) with lower coefficient of variation (33 *vs* 266 %).

**Conclusions:**

Aerobic storage of *O. ostertagi* eggs at 25 °C led to a significant increase in ITS2 copies after 12 h due to embryonation and subsequent hatching. In contrast, anaerobic storage (vacuum packing) at 25 °C completely inhibited egg development and any undesirable semi-quantification bias for up to 336 h. Hence, vacuum packing is an optimal storage strategy prior to molecular diagnostic analyses. Alternatively, aerobic storage at 4 °C for up to 72 h can be used. Due to high copy numbers and lower genetic variation, the L1 stage may be considered for diagnostics and further molecular research.

**Electronic supplementary material:**

The online version of this article (doi:10.1186/s13071-016-1657-4) contains supplementary material, which is available to authorized users.

## Background

Diagnosis of economically important strongylids [1] has traditionally relied on coproculture and differentiation of third-stage larvae (L3) which is laborious [2–4] and prone to low specificity and sensitivity [5, 6]. Furthermore, early diagnosis of anthelmintic resistance (AR) and monitoring of the species surviving anthelmintic treatment requires increasingly efficient methods [7]. In order to overcome these limitations and improve the diagnostic options, molecular methodologies have been developed for sensitive species identification of a number of important gastrointestinal nematodes of small ruminants [8–11] and cattle [12–14]. Recently, a real-time semi-quantitative polymerase chain reaction (qPCR) targeting the Internal Transcribed Spacer 2 (ITS2) of the ribosomal DNA (rDNA) was described for species-specific semi-quantification of two important strongylids of cattle, *Cooperia oncophora* and *Ostertagia ostertagi* [15]. As promising diagnostic tools, molecular methodologies must have all sources of bias documented throughout the complete diagnostic pipeline and in this context, storage of the samples can introduce bias due to rapid development of nematode eggs. Previous research has studied the effect of chemical preservation of strongylid eggs on qPCR results [16] but systematic and rigorous testing of the impact of anaerobic/aerobic storage and varying temperatures throughout time are lacking.

The aims of this study were: (i) to document and quantify how the development of *O. ostertagi* eggs affects ITS2 copies under a multitude of storage conditions, and (ii) to suggest optimal storage conditions for faecal samples/bovine nematode eggs in a diagnostic pipeline that involves detection and semi-quantification by molecular methodologies.

## Methods

### Experimental design

**Fig. 1:**
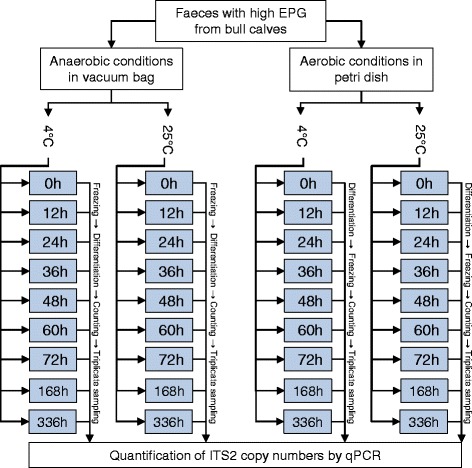
Schematic overview of the study design. Anaerobic samples (*n* = 9 × 2) contained 10 g of freshly recovered faeces with *Ostertagia ostertagi* eggs and stored in vacuum bags under anaerobic conditions. Samples were subjected to either 4 °C or 25 °C for duration of 0 to 336 h. To stop egg development the samples were frozen at -20 °C. Subsequently, *O. ostertagi* were isolated and differentiated to developmental stage. Finally, 25× *O. ostertagi* eggs or L1 were counted and transferred to a clean petri dish. This was done in triplicates of each sample and ITS2 copies were quantified by qPCR. Aerobic samples (*n* = 9 × 2) were produced identically to anaerobic samples but eggs were isolated from faeces before storage and differentiated immediately at each time point. A total of 108 biological replicates were subjected to qPCR semi-quantification. *Abbreviations*: EPG, eggs per gram; ITS2, Internal Transcribed Spacer 2; qPCR, real-time semi-quantitative polymerase chain reaction

**Fig. 2:**
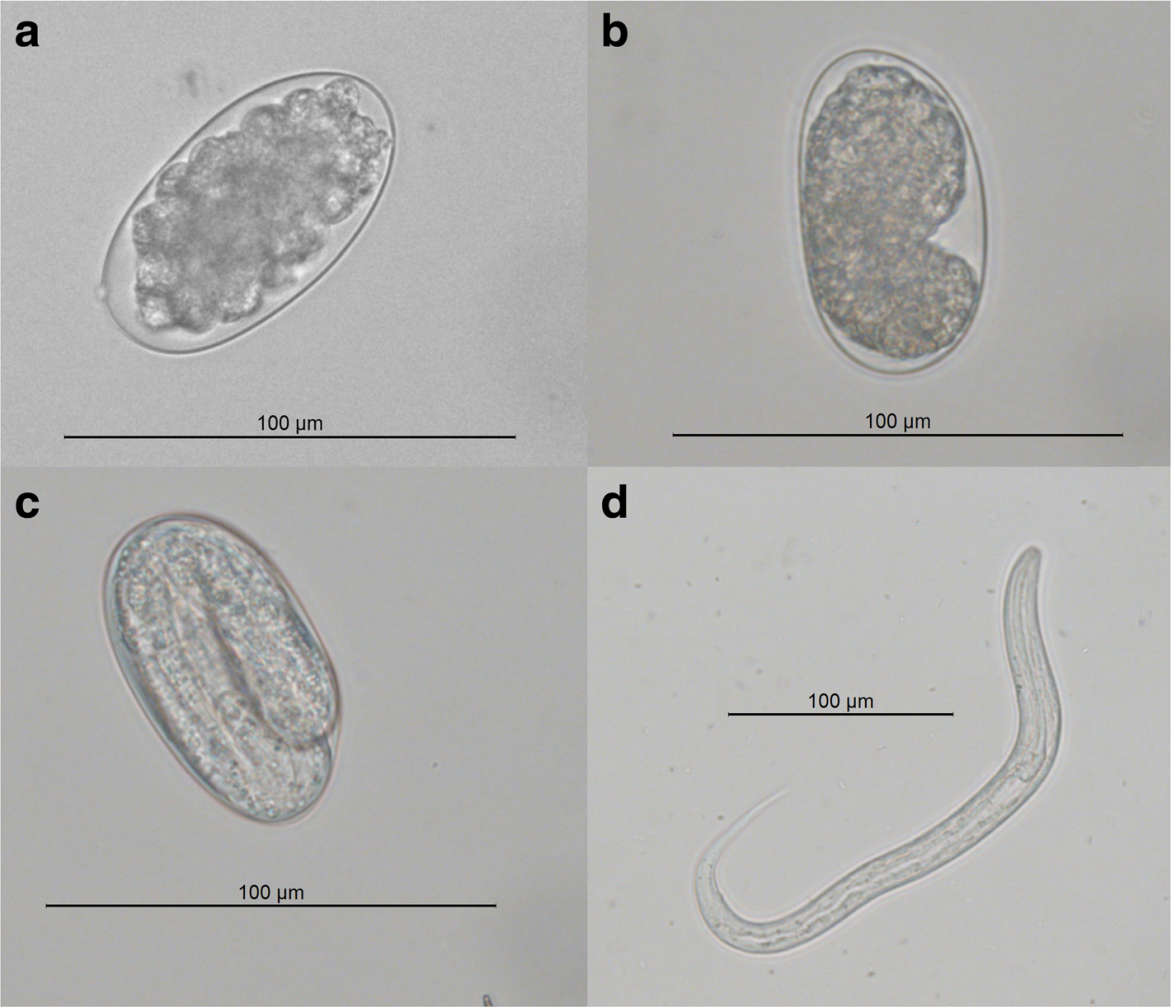
*Ostertagia ostertagi* in various stages of development. **a** Unembryonated egg. **b** Early embryonated egg. **c** Embryonated egg. **d** First-stage larvae, L1

Eggs of *Ostertagia ostertagi* were stored under anaerobic or aerobic conditions at 4 °C or 25 °C for durations of 0, 12, 24, 48, 60, 72, 168 and 336 h. Biological triplicate samples were analysed by qPCR (Fig. 1) and correlation between the stage of development (Fig. 2) and ITS2 copies was analysed.

### Setup of aerobic samples

*Ostertagia ostertagi* eggs were isolated from fresh faeces recovered from monospecies-infected calves by adding 26 ml water to 4 g of faeces which were mixed and sieved through gauze (type 28, BSN Medical, Hamburg, Germany) into two 15 ml Falcon tubes and centrifuged at 172 *g* for 10 min at 4 °C. The pellet was added 8 ml flotation fluid (saturated NaCl with 50 g glucose per 100 ml; density = 1.27 g/ml, [17]) and sieved again (20 μm, Buch & Holm A/S, Herlev, Denmark). The eggs were deposited in 9 × 2 Petri dishes containing 1–5 × 10^3^ eggs each. At each time point, eggs and/or L1 (*n* = 15) were differentiated (Fig. 2) and photographed at a magnification of 400× (DMR Type 020–525.024, Leica Microsystems, Wetzlar, Germany). Subsequently, to stop egg/larvae development, the Petri dishes were frozen at -20 °C for two weeks. After thawing, 25 *O. ostertagi* eggs or larvae were counted under a stereo microscope (Type M125, Leica Microsystems, Wetzlar, Germany) and transferred to a clean Petri dish. Three biological triplicates were produced for each time point.

### Setup of anaerobic samples

From the same faeces used for aerobic samples, aliquots of 10 g were immediately transferred to 9 × 2 individual plastic bags (ORVED, Musile di Piave, Italy). Anaerobic conditions were produced by a vacuum-sealer (Freshield Touch, CSE Co, Gyeonggi-do, Korea). Following storage for the specified periods, the bags were frozen at -20 °C for two weeks. Subsequently, the eggs were isolated, differentiated, photographed, and quantified to produce triplicate samples for qPCR as described for the aerobic samples.

### DNA extraction and semi-quantitative real-time PCR analysis

Biological triplicates of eggs and/or L1 were added 1,000 μl lysis buffer (Buffer ATL, QIAGEN, Hilden, Germany) and 20 μl of Proteinase K (20 mg/ml, QIAGEN), re-sealed with Parafilm® M (Bemis NA, Oshkosh, USA) and incubated at 56 °C for 2 h. After incubation, each sample was mixed by gentle shaking and DNA was extracted from 1/5 of the total lysis buffer (200 μl) and processed by QIAamp® DNA Mini Kit (QIAGEN) following the manufacturer’s instructions. Primers and probe targeted a 91 bp stretch in the *O. ostertagi* ITS2 sequence (GenBank® accession no. AB245021.2) from position 1036 according to Höglund et al. [15]. Duplicate amplifications were performed with a Rotor-gene Q RG-6000® (QIAGEN) in total volumes of 25 μl using 0.65 U Taq2000® polymerase (Agilent Technologies, Santa Clara, CA, USA), 0.3 μM of forward and reverse primer, 0.2 μM probe, 200 μM dNTP and 5.5 mM MgCl_2_ using 2 μl DNA as template. The cycling conditions were 95 °C for 10 min and amplification for 50 cycles (95 °C for 15 s, 62 °C for 60 s). Semi-quantifications were performed by extrapolating cycle threshold (Ct) values to a standard curve with 2 × 10^7^, 2 × 10^6^, 10^5^, 10^4^ and 10^3^ molecules μl^−1^ of a plasmid construct comprising the *O. ostertagia* ITS2 sequence according to Höglund et al. [15]. Positive and negative DNA controls and a water template control were included for each run.

### Statistical analysis

Statistical analyses were performed using GraphPad Prism® version 5.02 for Windows (GraphPad Software, La Jolla, CA, USA) and R Commander version 2.0–4 [18]. The temporal effect on ITS2 copies was analysed by one-way analysis of variance (ANOVA) on the log transformed ITS2 copies with Dunnett’s *post-hoc* test with 0 h as the baseline. ITS2 copies were grouped, either uncorrected or corrected for DNA extraction partitioning and compared with the Kruskal-Wallis non-parametric test followed by Dunn’s multiple comparison *post-hoc* test.

## Results

### Storage conditions and ITS2 copies

**Fig. 3:**
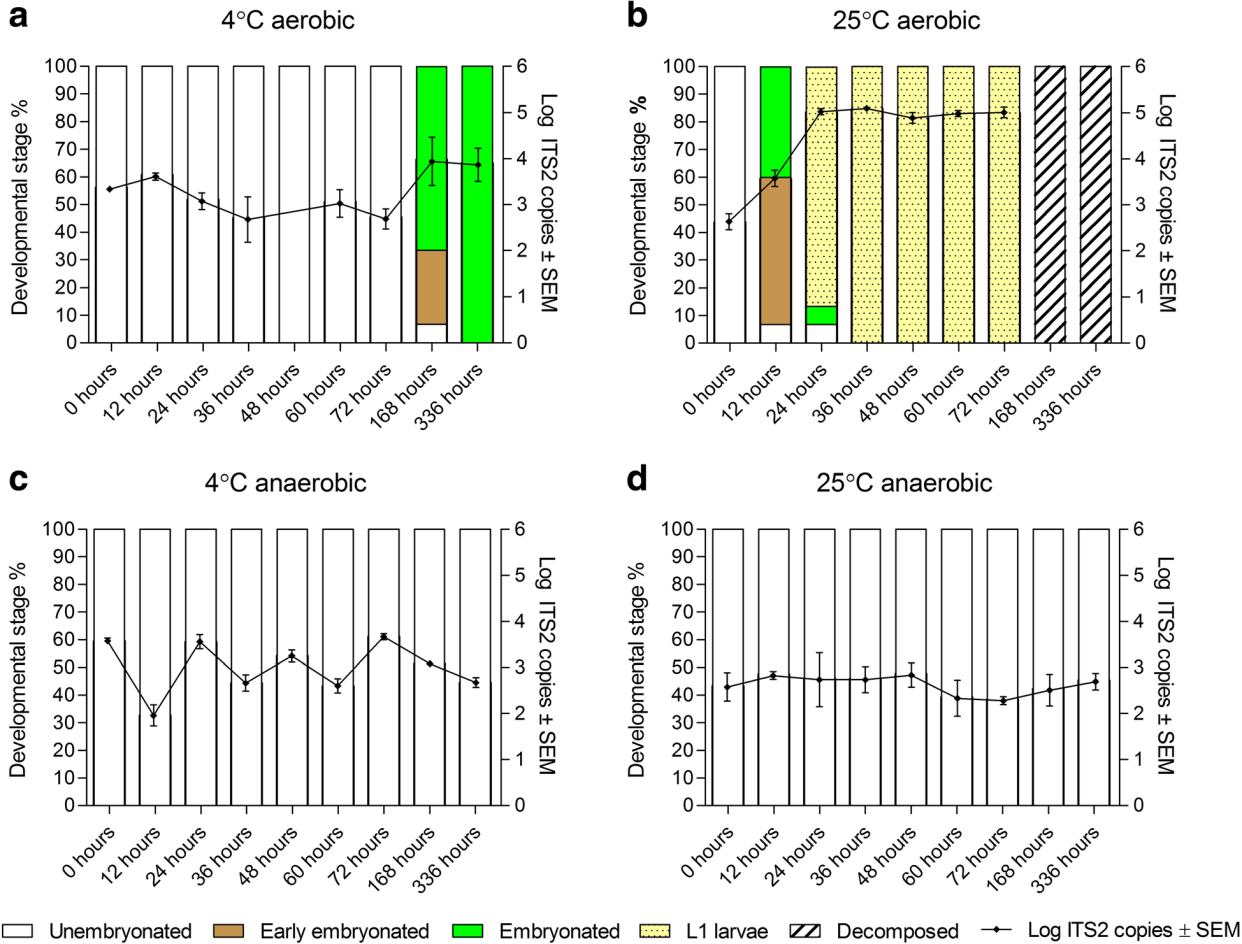
Log-transformed ITS2 copies plotted against developmental stages of *Ostertagia ostertagi* throughout the four sub-experiments. **a** 4 °C under aerobic conditions. **b** 25 °C under aerobic conditions. **c** 4 °C under anaerobic conditions. **d** 25 °C under anaerobic conditions. The X-axis indicates storage time. Distributions (%) of developmental stages are plotted on left Y-axis (*white bar*: unembryonated eggs; *brown bar*: early embryonated eggs; *green bar*: embryonated eggs; *yellow dotted bar*: L1 larvae; *shaded bar*: decomposed parasites). Developmental stage was determined from photographs of 15 randomly selected eggs from each of nine time points. Log-transformed ITS2 copies are plotted on right Y-axis and are indicated by a *black line*. ITS2 copies were obtained from qPCR analysis of biological triplicate samples containing DNA from 25 parasites (eggs or larvae). Error bars indicate standard error of the mean (SEM) of log-transformed ITS2 copies

Under aerobic conditions at 4 °C, there was no significant temporal effect on ITS2 copies (ANOVA, *F*_(7,16)_ = 2.365, *P* = 0.07) which ranged from a mean (± SEM) of 2,381 ± 196 ITS2 copies at 0 h to 16,191 ± 12,663 at 336 h. Microscopic examination revealed that 67 % of the eggs were embryonated after 168 h and 100 % after 336 h (Fig. 3a). At the 48 h time point, the eggs were lost prior to qPCR due to a human error. At 25 °C, there was a significant temporal effect due to embryonation and subsequent hatching on ITS2 copies (*F*_(6,14)_ = 66.84, *P* < 0.0001) which increased from 482 ± 184 ITS2 copies at 0 h to 106,707 ± 29,396 at 72 h. *Post-hoc* testing revealed significant differences from 12 h of storage and onwards, compared to the 0 h group (all *P* < 0.0001). Microscopic examination showed 87 % L1 after 24 h and 100 % L1 after 36 h (Fig. 3b). At 168 and 336 h, all L1 were decomposed and unsuitable for exact counting.

Under anaerobic conditions, there was a significant temporal effect on ITS2 copies at 4 °C (F_(8,18)_ = 17.31, *P* < 0.0001) ranging from 3,802 ± 508 ITS2 copies at 0 h to 496 ± 121 at 336 h. However, ITS2 copies at 24, 48, 72 and 168 h were not significantly different from 0 h in the *post-hoc* tests (*P* > 0.05), and no morphological changes were observed (Fig. 3c).

No significant temporal effect was observed at 25 °C under anaerobic conditions (F_(8,18)_ = 0.41, *P* = 0.90), and the ITS2 copies ranged from 576 ± 244 ITS2 copies at 0 h to 603 ± 187 at 336 h. Correspondingly, no morphological changes were observed throughout the 336 h of storage at 25 °C under anaerobic conditions (Fig. 3d). The full collection of photographs demonstrating the morphological development in detail is presented in Additional file 1: Figures S1-S34.

### Level of development and ITS2 copies

**Fig. 4:**
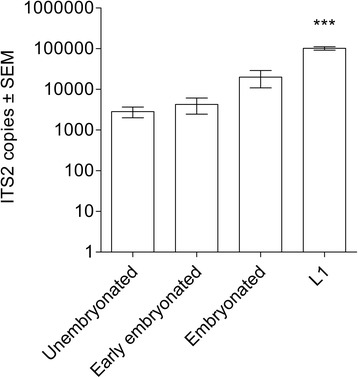
ITS2 copies of four developmental stages of *Ostertagia ostertagi*. Sample mean of ITS2 copies (molecules μl^-1^) illustrated on the Y-axis plotted against each developmental stage (X-axis). Error bars indicate standard error of the mean (SEM). Asterisks denote statistical significance: *** = *P* < 0.0001

Analysis of developmental stages (Table 1) revealed that the number of ITS2 copies of L1 was significantly different from those of the other three developmental stages (*H* = 39.99, *P* < 0.0001), ranging from 2,841 ± 840 ITS2 copies for unembryonated eggs to 101,767 ± 9,753 for L1 (Fig. 4). Comparison of mean ITS2 copies corrected for genomic DNA partitioning revealed that a single unembryonated egg contained 568 ± 168 ITS2 copies compared to 20,353 ± 1,950 ITS2 copies detected in one L1 larva. The coefficient of variation ranged from 266 % for the unembryonated eggs to 33 % for hatched L1 (Table 1). The groups “early embryonated eggs” and “embryonated eggs” comprised 54 % and 84 % of the designated stages, whereas “unembryonated eggs” and “L1” comprised 100 %.

**Table 1:** ITS2 copies obtained from four stages of development of *Ostertagia ostertagi*

	Unembryonated eggs	Early embryonated eggs	Embryonated eggs	First-stage larvae (L1)
	(*n* = 81)	(*n* = 3)	(*n* = 6)	(*n* = 12)
Mean sample ± SEM (molecules μl^-1^)^a^	2,841 ± 840	4,285 ± 1,818	19,970 ± 9,090	101,767 ± 9,753
Mean parasite ± SEM (molecules)^b^	114 ± 34	171 ± 73	799 ± 364	4,070 ± 390
Mean parasite corr. ± SEM (molecules)^c^	568 ± 168	857 ± 363	3,994 ± 1,818	20,353 ± 1,950
Coefficient of variation (%)	266	73	111	33

^**a**^Mean sample = total number of ITS2 copies for a given stage divided by the number of samples

^**b**^Mean parasite = mean sample divided by 25× parasites

^**c**^Mean parasite corr. = theoretical value of the mean parasite multiplied by five to correct for genomic DNA partitioning. Each sample contained 25× parasites

Abbreviations: *n* number of samples analyzed for each developmental stage, *SEM* standard error of the mean

## Discussion

This study showed that anaerobic storage of *O. ostertagia* eggs at room temperature for up to 336 h completely inhibited any effects on morphology and qPCR semi-quantifications. The inhibited development is in agreement with previous investigations on the closely related ruminant trichostrongylid *Cooperia oncophora* [19] and horse strongylids [20, 21], but to our knowledge we are the first to document the efficiency of anaerobic storage in the context of an extremely sensitive molecular diagnostic pipeline. Refrigeration at aerobic conditions suppressed egg development for up to 72 h, which is also in agreement with previous findings [22–24], but at room temperature a significant increase in ITS2 copies was observed already from 12 h. Our findings warrant consideration whenever faecal samples are stored prior to qPCR for quantification of species-specific parasite loads such as evaluation of drug efficiency, epidemiological research and diagnosis of parasitic infections in both animals and humans.

A substantial variation in ITS2 copies was observed in our study, even under anaerobic conditions. Some of this variation in the qPCR outcome could relate to genetic variation in the target sequence of the forward primer, which may provide suboptimal conditions for the qPCR. Indeed, a BLAST® search on the targeted ITS2 sequence (GenBank accession no: AB245021.2, query range 1,036–1,126 bp) revealed a genetic variability of 1–2 single nucleotide polymorphisms (SNPs) in the forward primer region among 31 % of the available *O. ostertagi* ITS2 sequences (Additional file 2). The variation is observed in isolates of diverse geographical origin but a population genetic analysis of the ITS2 is beyond the scope of this paper.

Another possible source of variation includes inconsistent extraction efficiency of genomic DNA from eggs compared to L1. In order to ensure consistent extraction efficiency the protocol was validated prior to the study using known numbers of eggs and L1 that were subjected to various concentrations of proteinase K and incubation times. Following homogenisation, the samples were evaluated microscopically for the presence of parasite fragments, which could not be detected in any of the samples thus indicating adequate homogenisation. Finally, 2-fold variations in ribosomal copy numbers have been found in *Caenorhabditis elegans* propagated by repeated population bottlenecking [25]; a similar situation may have arisen in the *O. ostertagi* populations over time.

Ideally, more biological replicates should have been included to avoid statistical noise caused by genetic variability. Yet, addition of more replicates may have introduced another source of variation due to egg development during sample preparation. The current study design comprised 36 unique trials, distributed on four storage conditions and analysed in biological triplicates at nine time points. This resulted in a total of 108 biological samples, which were quantified in technical duplicates. Supplementary samples would have necessitated additional manpower, which would have added further variation. Moreover, it would be relevant to repeat the study with other species or genera of gastrointestinal nematodes e.g. *C. oncophora* to confirm the consistency of the findings*.* Despite the limitations of our study, the conclusions can be clearly inferred from the genetic and morphological data.

Under aerobic conditions, morphological development and a trend towards increased ITS2 copies were observed after 168 h and 336 h thus stressing the importance of quick sample turnover time even at cold storage. At 25 °C, the optimal developmental temperature of *O. ostertagi* (23–25 °C) [26] and other ruminant [19, 27] and equine nematodes [21], a clear and significant increase in ITS2 copies was observed already from 12–72 h. These findings justify re-evaluation of diagnostic procedures in parasitological laboratories where samples are stored under ambient aerobic conditions as part of a molecular diagnostic pipeline. Surprisingly, anaerobic storage at 4 °C was found to have a significant temporal effect on ITS2 copies (drop at 336 h), but considering the lack of morphological changes these results were probably due to intrinsic variations caused by the mentioned ITS2 variability. No significant temporal effects on ITS2 copies or morphology were observed following anaerobic storage at 25 °C. Furthermore, less molecular variation was observed at 25 °C regardless of storage period. We hypothesise that this finding is related to the microenvironment in the faeces. Warm conditions support microbial activity which in turn might reduce the presence of faecal PCR inhibitors resulting in less variation in ITS2 copies at higher temperatures. This result may have important practical consequences as transport of faecal samples from farm to laboratory is far more practical at room temperature than cold transport.

In order to create anaerobic conditions, faeces containing *O. ostertagi* eggs were vacuum-packed using an easily available kitchen machine. While purified *O. ostertagi* eggs will quickly disrupt due to pressure if they are vacuum-packed (data not shown), storage of eggs in faeces was essential to secure the integrity of the parasites and maintain a realistic evaluation of the vacuum packing strategy. Thus, faeces were only present during storage of the anaerobic samples but not during storage of aerobic samples, and therefore the outcome may potentially have been influenced by other factors such as e.g. pH and humidity rather than oxygen tension. Incorporation of an inhibition control by spiking a non-related target to the samples could confirm any presence of potential inhibitors.

In summary, we found that oxygen clearly outranks temperature in the hierarchy of bionomic requirements of *O. ostertagi*. This is of less importance if the aim of the analysis is purely qualitative, or if the samples to be compared have been stored identically under controlled laboratory conditions. However, field samples may be subject to a wide range of storage times and temperatures, and in such cases our results strongly advocate the use of vacuum packing as sole storage strategy for faecal samples intended for semi-quantitative molecular analysis. This is in accordance with the recommendations in the World Association for the Advancement of Veterinary Parasitology (WAAVP) guidelines [28], which now have robust evidence of the superiority of vacuum packing prior to molecular diagnostics. A disadvantage of effective vacuum packing is the requirement of electricity, which is not always at hand in field settings. In such cases, chemical preservation may be an alternative strategy [16] but validation of other common preservatives such as ethanol is still lacking. Ultimately, the strategy for creating anaerobic conditions should be as easy as possible to allow consistent and adequate routine sampling.

The first-stage larvae (L1) of *O. ostertagi* exhibited ~ 36 times higher copy numbers (20,353 ± 1,950) than unembryonated eggs (568 ± 168; *P* < 0.0001) with lower coefficient of variation (33 *vs* 266 %). Consequently, this parasitic stage may offer an attractive alternative for sensitive, semi-quantitative diagnostics, which is particularly relevant in cattle due to frequent low level infections [29]. The L1 larval stage can be cultivated within 24 h, with developmental efficacies of over 80 % and similar hatching rates regardless of strongylid species [3, 11].

## Conclusions

Aerobic storage of *O. ostertagi* eggs at 25 °C led to a significant increase in ITS2 copies from 12 h due to embryonation and subsequent hatching. In contrast, anaerobic storage (vacuum packing) at 25 °C completely inhibited egg development and any undesirable semi-quantification bias for up to 336 h. Hence, vacuum packing is an optimal storage strategy prior to molecular diagnostic analyses. Alternatively, aerobic storage at 4 °C for up to 72 h can be used. Due to high copy numbers and less genetic variation, the L1 stage may be considered for diagnostics and further molecular research.

## Abbreviations

ANOVA, one-way analysis of variance; AR, anthelmintic resistance; Ct, cycle threshold; DNA, deoxyribonucleic acid; dNTP, deoxyribonucleoside triphosphate; ITS2, Internal Transcribed Spacer 2; kb, Kilobases; L1, first-stage larvae; L3, third-stage larvae; MgCl_2_, magnesium chloride; NaCl, sodium chloride; PCR, polymerase chain reaction; qPCR, real-time semi-quantitative polymerase chain reaction; rDNA, ribosomal DNA; SEM, standard error of the mean; SNP, single nucleotide polymorphisms; U, units; WAAVP, World Association for the Advancement of Veterinary Parasitology

## Additional files

Additional file 1: Figures S1-S34. Photomicrographs showing egg development under aerobic and anaerobic storage at either 4 °C or 25 °C (magnification of 400×). Figure S1. Aerobic storage at 4 °C after 0 h. Figure S2. Aerobic storage at 4 °C after 12 h. Figure S3. Aerobic storage at 4 °C after 24 h. Figure S4. Aerobic storage at 4 °C after 36 h. Figure S5. Aerobic storage at 4 °C after 48 h. Figure S6. Aerobic storage at 4°C after 60 h. Figure S7. Aerobic storage at 4 °C after 72 h. Figure S8. Aerobic storage at 4 °C after 168 h. Figure S9. Aerobic storage at 4 °C after 336 h. Figure S10. Aerobic storage at 25 °C after 0 h. Figure S11. Aerobic storage at 25 °C after 12 h. Figure S12. Aerobic storage at 25 °C after 24 h. Figure S13. Aerobic storage at 25 °C after 36 h. Figure S14. Aerobic storage at 25 °C after 48 h. Figure S15. Aerobic storage at 25 °C after 60 h. Figure S16. Aerobic storage at 25 °C after 72 h. Figure S17. Anaerobic storage at 4 °C after 0 h. Figure S18. Anaerobic storage at 4 °C after 12 h. Figure S19. Anaerobic storage at 4°C after 24 h. Figure S20. Anaerobic storage at 4 °C after 36 h. Figure S21. Anaerobic storage at 4 °C after 48 h. Figure S22. Anaerobic storage at 4 °C after 60 h. Figure S23. Anaerobic storage at 4 °C after 72 h. Figure S24. Anaerobic storage at 4 °C after 168 h. Figure S25. Anaerobic storage at 4 °C after 336 h. Figure S26. Anaerobic storage at 25 °C after 0 h. Figure S27. Anaerobic storage at 25 °C after 12 h. Figure S28. Anaerobic storage at 25 °C after 24 h. Figure S29. Anaerobic storage at 25 °C after 36 h. Figure S30. Anaerobic storage at 25 °C after 48 h. Figure S31. Anaerobic storage at 25 °C after 60 h. Figure S32. Anaerobic storage at 25 °C after 72 h. Figure S33. Anaerobic storage at 25 °C after 168 h. Figure S34. Anaerobic storage at 25 °C after 336 h. (PDF 17635 kb) Additional file 2: Alignment of 49 ITS2 sequences from *Ostertagia ostertagi* obtained from BLAST on the *O. ostertagi* ITS2 sequence (GenBank AB245021.2|: 1,036–1,126 bp) targeted by the qPCR developed by Höglund et al. [15] and used in this study. Primers and probe are aligned in the bottom. Asterisks indicate the complete nucleotide conservation throughout all sequences; 15 sequences (31 %) contained single nucleotide polymorphisms (SNPs) in the forward primer region. (DOC 35 kb)
