# Supplementary material for: The level of embryonation influences detection of Ostertagia ostertagi eggs by semi-quantitative PCR
Source: Parasit Vectors. 2016 Jun 29;9:368. doi: 10.1186/s13071-016-1657-4 (PMC4928311; doi:10.1186/s13071-016-1657-4)
Supplement: Additional file 1: Figures S1-S34. — Photomicrographs showing egg development under aerobic and anaerobic storage at either 4 °C or 25 °C (magnification of 400×). Figure S1. Aerobic storage at 4 °C after 0 h. Figure S2. Aerobic storage at 4 °C after 12 h. Figure S3. Aerobic storage at 4 °C after 24 h. Figure S4. Aerobic storage at 4 °C after 36 h. Figure S5. Aerobic storage at 4 °C after 48 h. Figure S6. Aerobic storage at 4°C after 60 h. Figure S7. Aerobic storage at 4 °C after 72 h. Figure S8. Aerobic storage at 4 °C after 168 h. Figure S9. Aerobic storage at 4 °C after 336 h. Figure S10. Aerobic storage at 25 °C after 0 h. Figure S11. Aerobic storage at 25 °C after 12 h. Figure S12. Aerobic storage at 25 °C after 24 h. Figure S13. Aerobic storage at 25 °C after 36 h. Figure S14. Aerobic storage at 25 °C after 48 h. Figure S15. Aerobic storage at 25 °C after 60 h. Figure S16. Aerobic storage at 25 °C after 72 h. Figure S17. Anaerobic storage at 4 °C after 0 h. Figure S18. Anaerobic storage at 4 °C after 12 h. Figure S19. Anaerobic storage at 4°C after 24 h. Figure S20. Anaerobic storage at 4 °C after 36 h. Figure S21. Anaerobic storage at 4 °C after 48 h. Figure S22. Anaerobic storage at 4 °C after 60 h. Figure S23. Anaerobic storage at 4 °C after 72 h. Figure S24. Anaerobic storage at 4 °C after 168 h. Figure S25. Anaerobic storage at 4 °C after 336 h. Figure S26. Anaerobic storage at 25 °C after 0 h. Figure S27. Anaerobic storage at 25 °C after 12 h. Figure S28. Anaerobic storage at 25 °C after 24 h. Figure S29. Anaerobic storage at 25 °C after 36 h. Figure S30. Anaerobic storage at 25 °C after 48 h. Figure S31. Anaerobic storage at 25 °C after 60 h. Figure S32. Anaerobic storage at 25 °C after 72 h. Figure S33. Anaerobic storage at 25 °C after 168 h. Figure S34. Anaerobic storage at 25 °C after 336 h. (PDF 17635 kb) [file 13071_2016_1657_MOESM1_ESM.pdf]

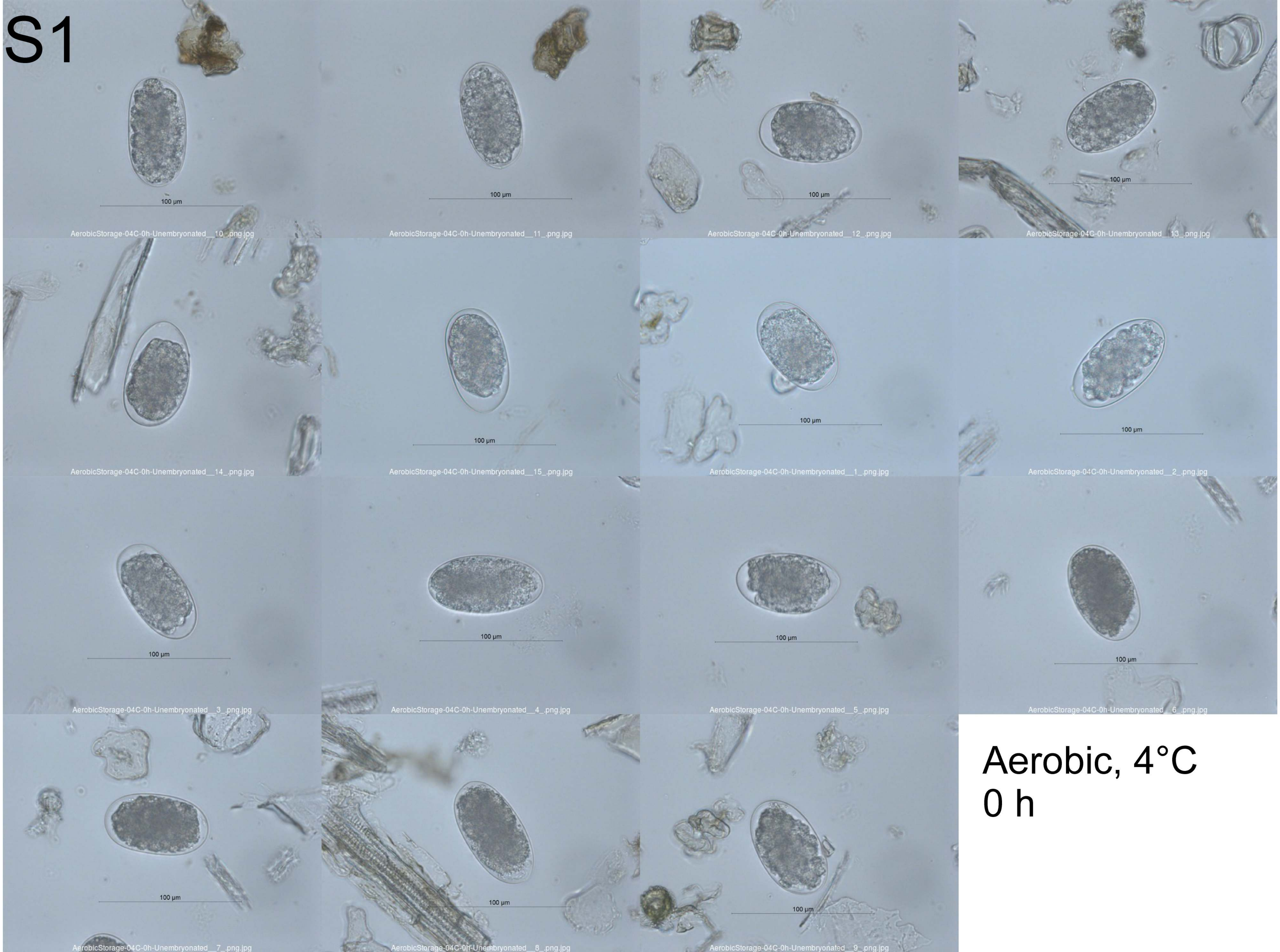
S1
100 µm
AerobicStorage-04C-0h-Unembryonated__10_.png.jpg
100 µm
AerobicStorage-04C-0h-Unembryonated__11_.png.jpg
100 µm
AerobicStorage-04C-0h-Unembryonated__12_.png.jpg
100 µm
AerobicStorage-04C-0h-Unembryonated__13_.png.jpg
AerobicStorage-04C-0h-Unembryonated__14_.png.jpg
100 µm
AerobicStorage-04C-0h-Unembryonated__15_.png.jpg
100 µm
AerobicStorage-04C-0h-Unembryonated__1_.png.jpg
100 µm
AerobicStorage-04C-0h-Unembryonated__2_.png.jpg
100 µm
AerobicStorage-04C-0h-Unembryonated__3_.png.jpg
100 µm
AerobicStorage-04C-0h-Unembryonated__4_.png.jpg
100 µm
AerobicStorage-04C-0h-Unembryonated__5_.png.jpg
100 µm
AerobicStorage-04C-0h-Unembryonated__6_.png.jpg
100 µm
AerobicStorage-04C-0h-Unembryonated__7_.png.jpg
100 µm
AerobicStorage-04C-0h-Unembryonated__8_.png.jpg
100 µm
AerobicStorage-04C-0h-Unembryonated__9_.png.jpg
Aerobic, 4°C
0 h

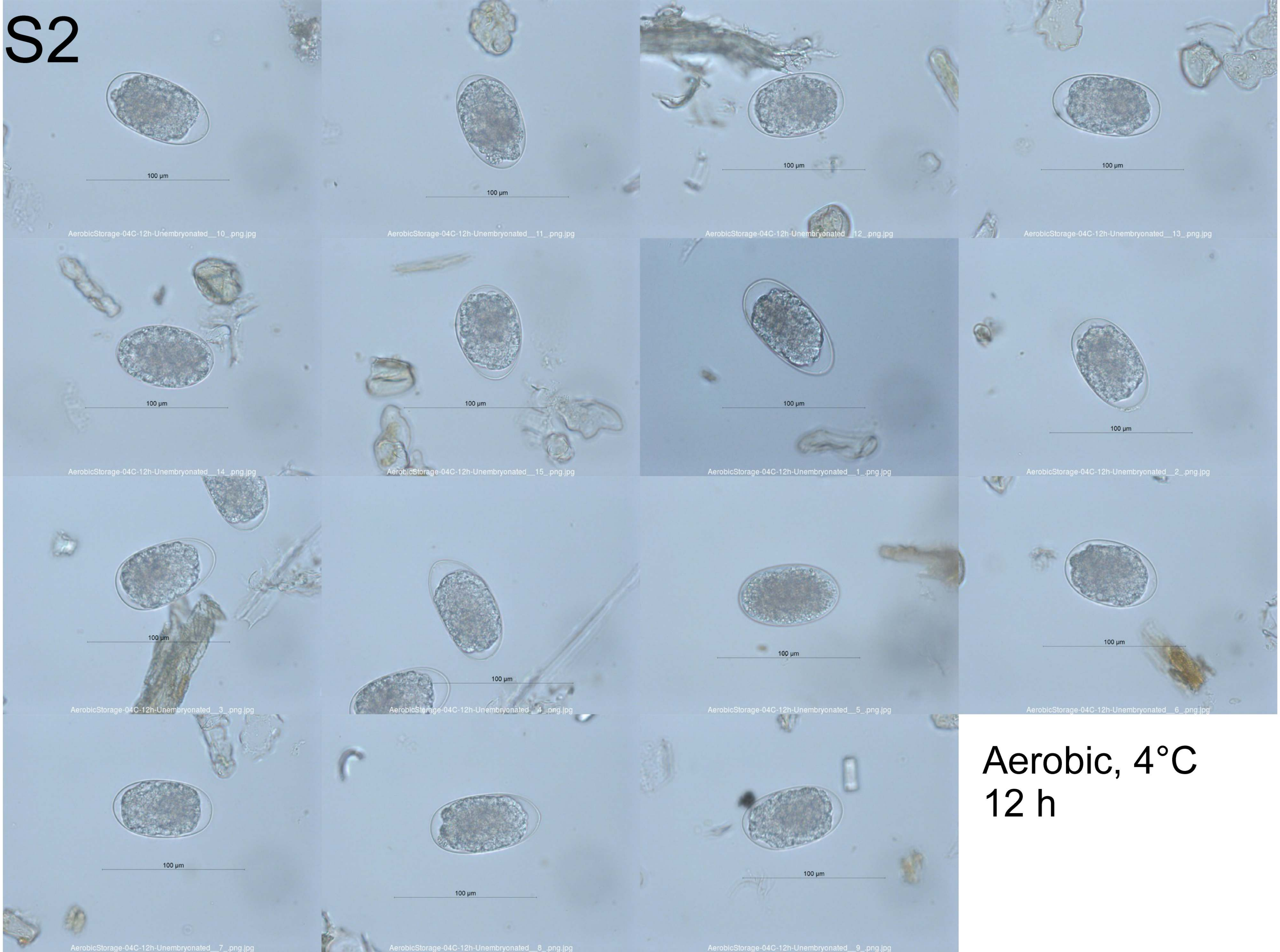
S2
100 µm
AerobicStorage-04C-12h-Unembryonated__10_.png.jpg
100 µm
AerobicStorage-04C-12h-Unembryonated__11_.png.jpg
100 µm
AerobicStorage-04C-12h-Unembryonated__12_.png.jpg
100 µm
AerobicStorage-04C-12h-Unembryonated__13_.png.jpg
100 µm
AerobicStorage-04C-12h-Unembryonated__14_.png.jpg
100 µm
AerobicStorage-04C-12h-Unembryonated__15_.png.jpg
100 µm
AerobicStorage-04C-12h-Unembryonated__1_.png.jpg
100 µm
AerobicStorage-04C-12h-Unembryonated__2_.png.jpg
100 µm
AerobicStorage-04C-12h-Unembryonated__3_.png.jpg
100 µm
AerobicStorage-04C-12h-Unembryonated__4_.png.jpg
100 µm
AerobicStorage-04C-12h-Unembryonated__5_.png.jpg
100 µm
AerobicStorage-04C-12h-Unembryonated__6_.png.jpg
100 µm
AerobicStorage-04C-12h-Unembryonated__7_.png.jpg
100 µm
AerobicStorage-04C-12h-Unembryonated__8_.png.jpg
100 µm
AerobicStorage-04C-12h-Unembryonated__9_.png.jpg
Aerobic, 4°C
12 h

S3

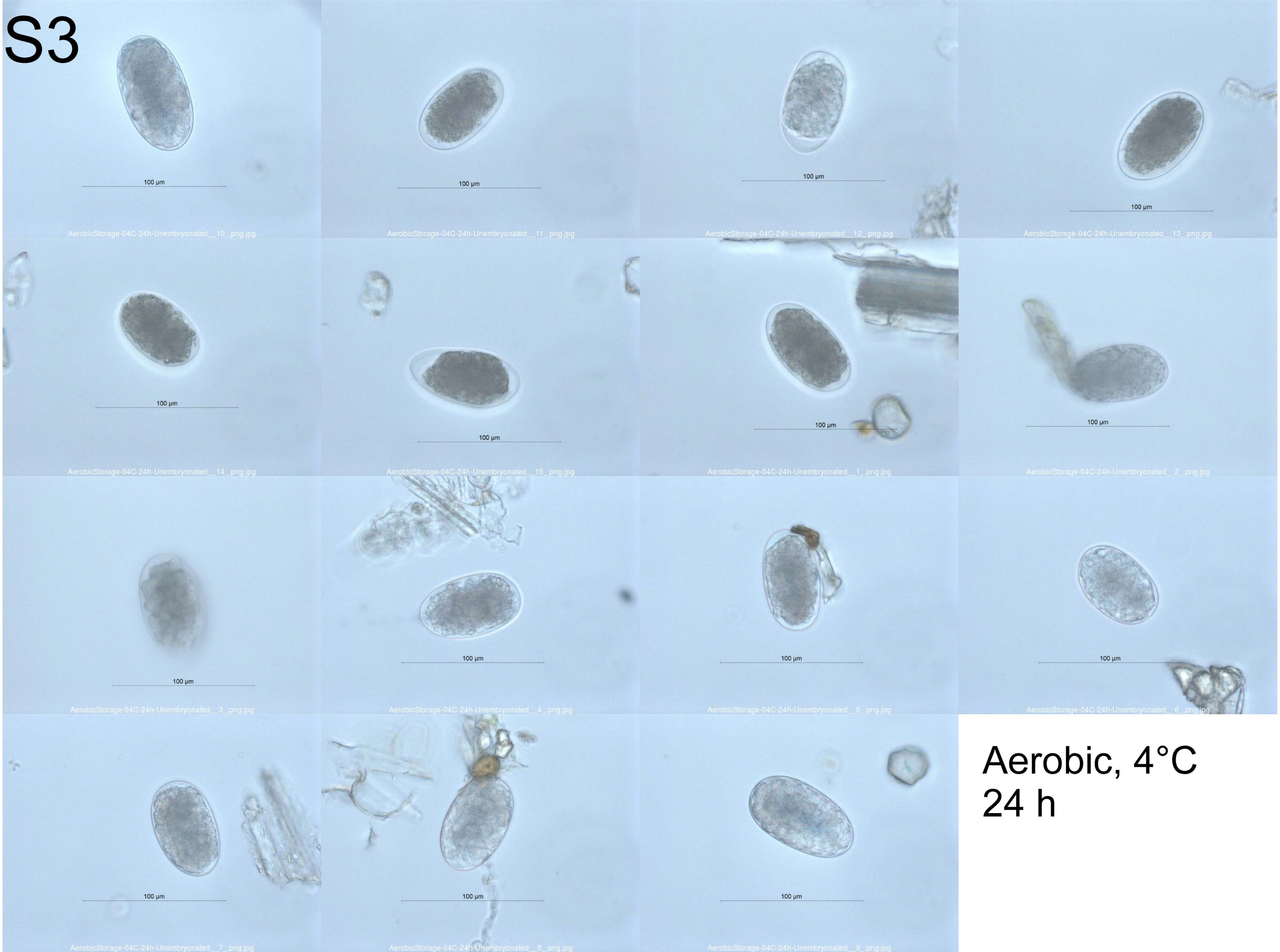

Aerobic, 4°C
24 h

S4

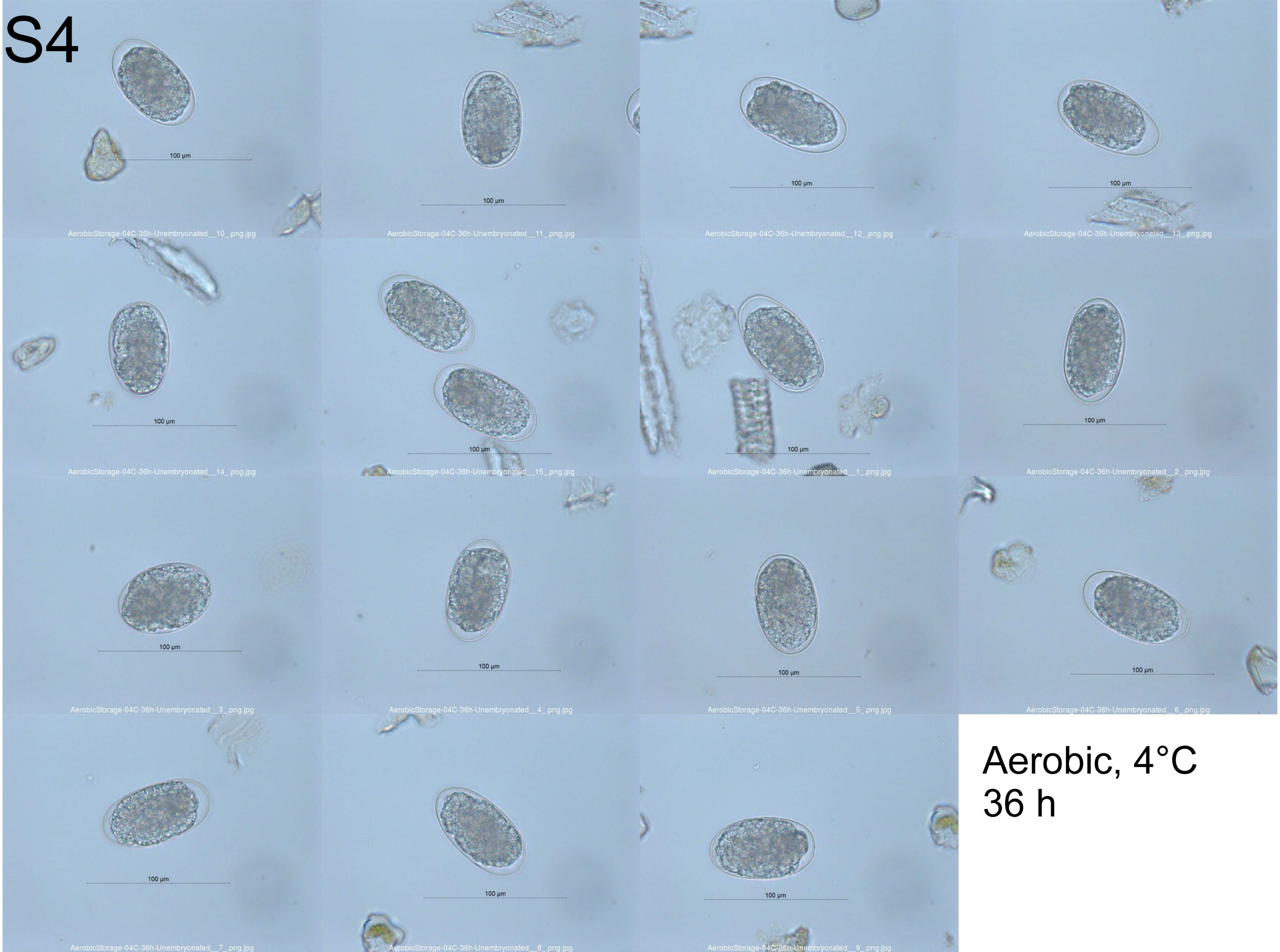

Aerobic, 4°C
36 h

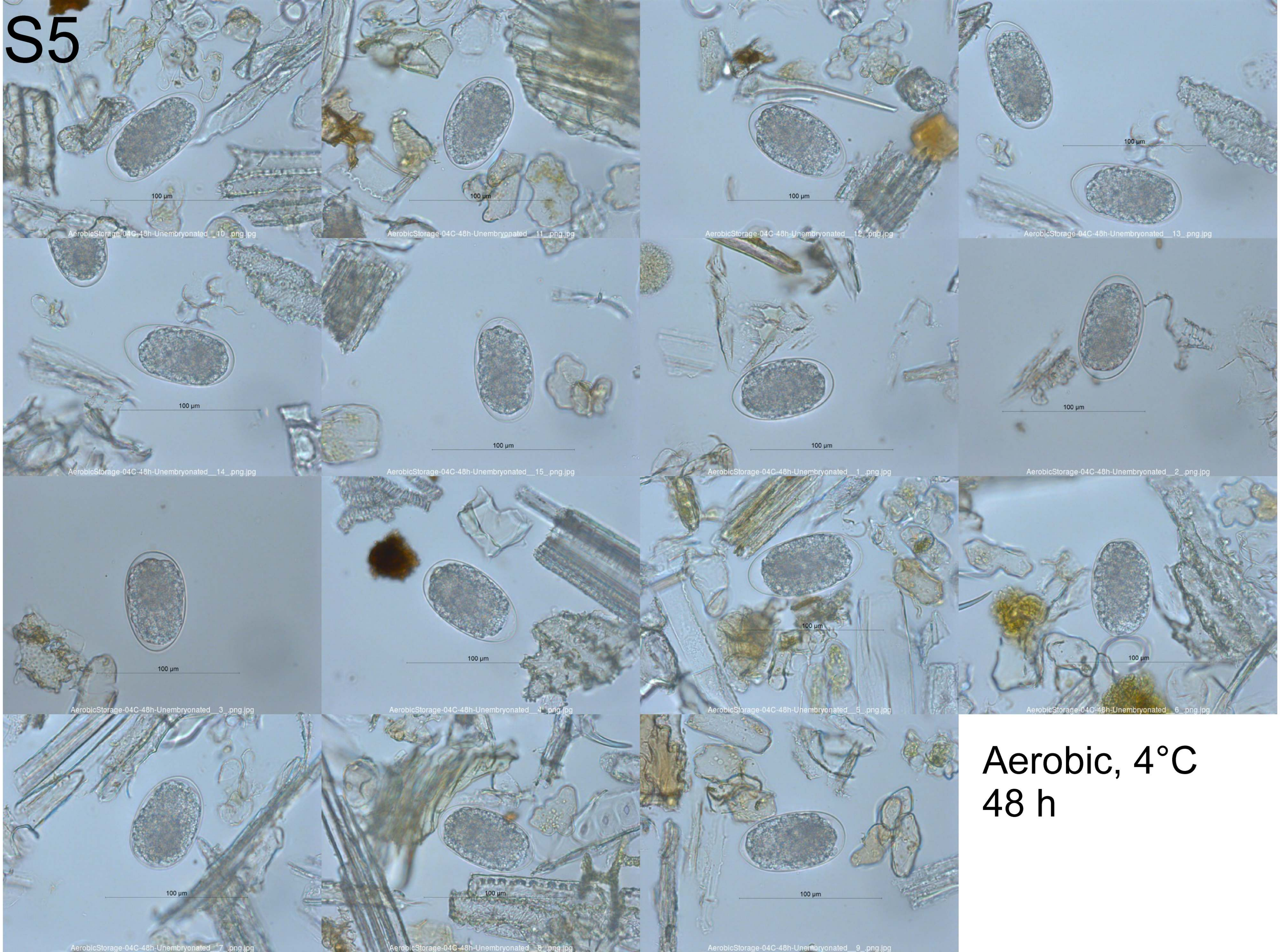

Aerobic, 4°C
48 h

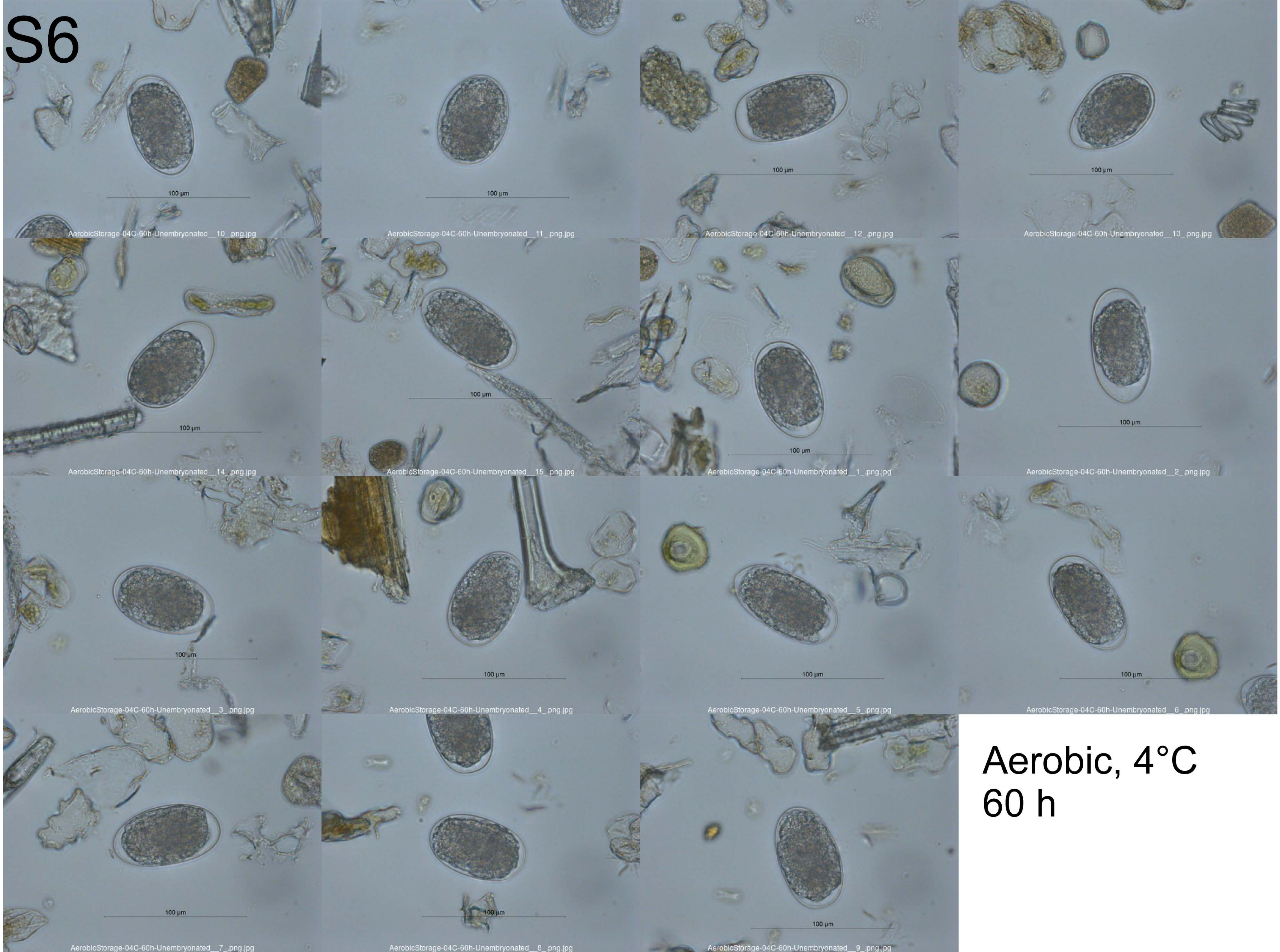

Aerobic, 4°C
60 h

S7

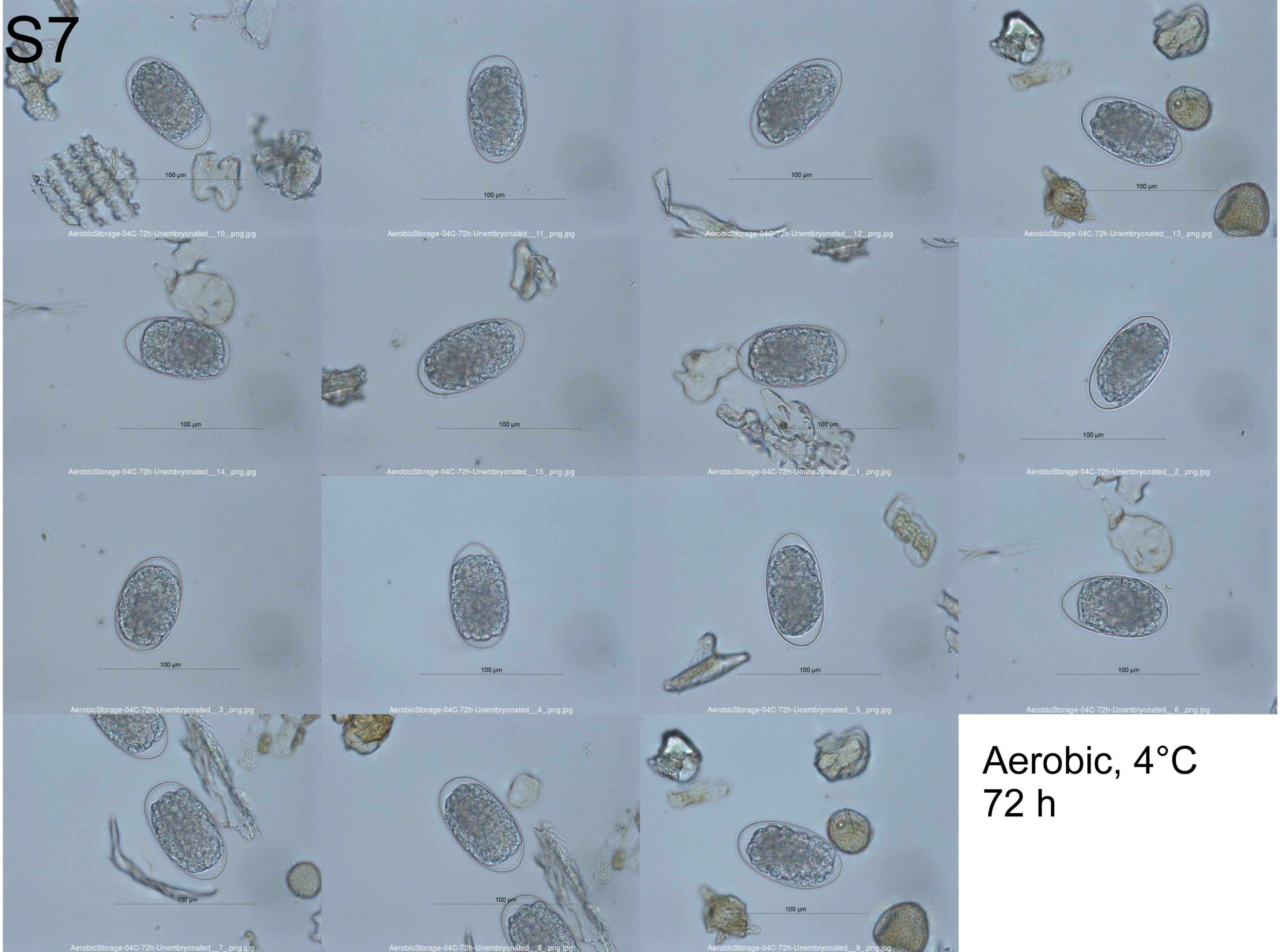

Aerobic, 4°C
72 h

S8

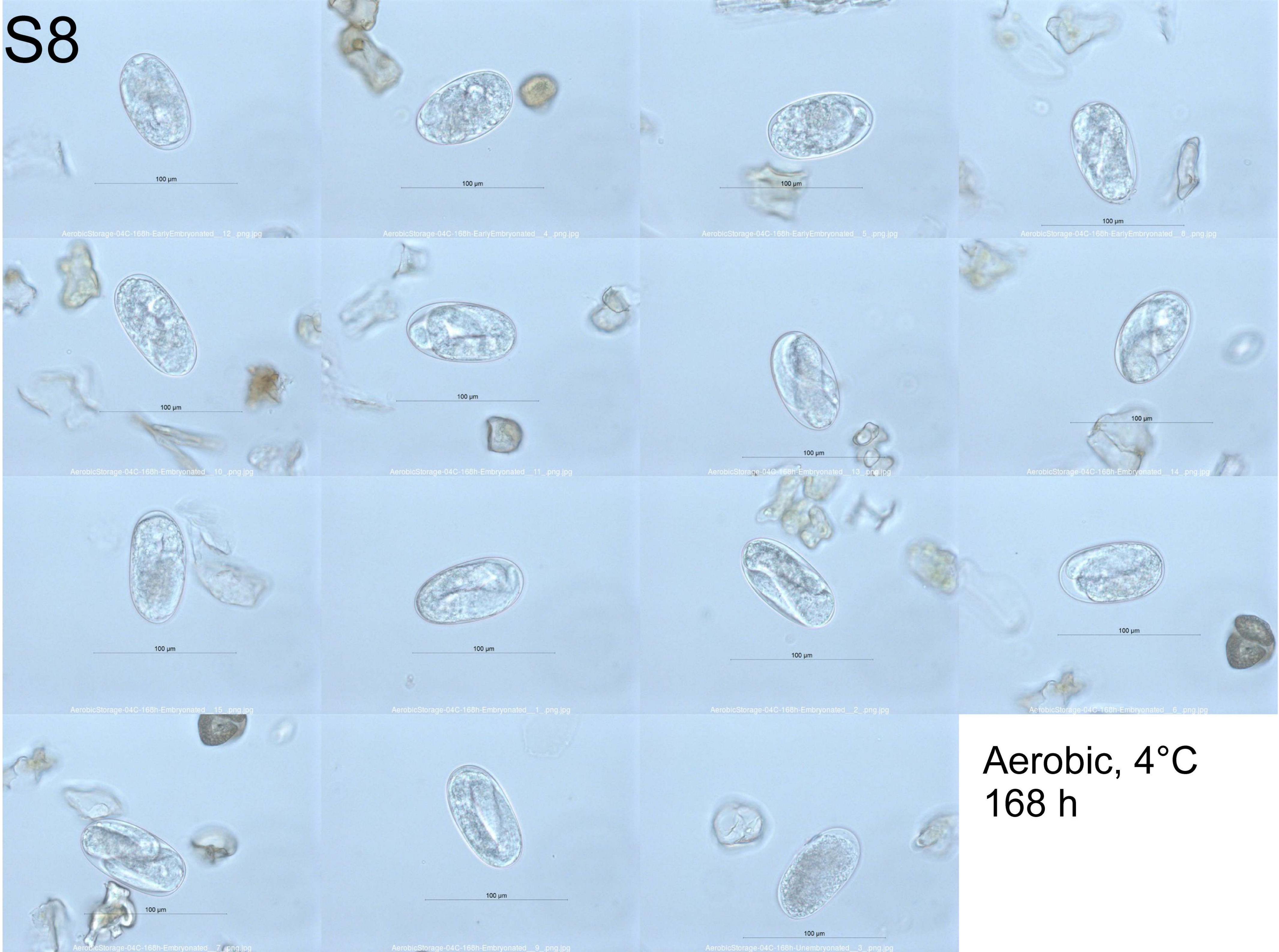

Aerobic, 4°C
168 h

S9

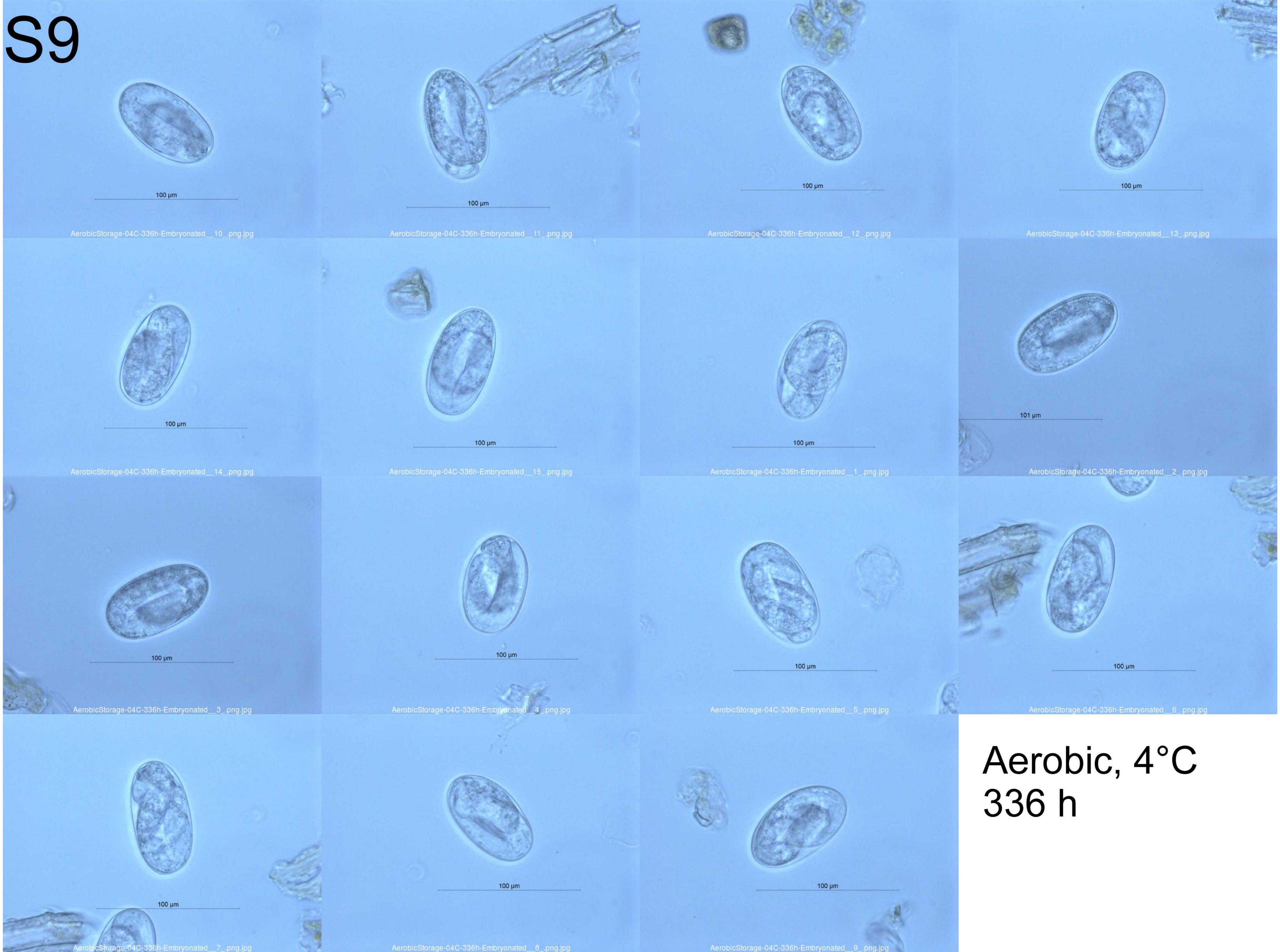

Aerobic, 4°C
336 h

S10

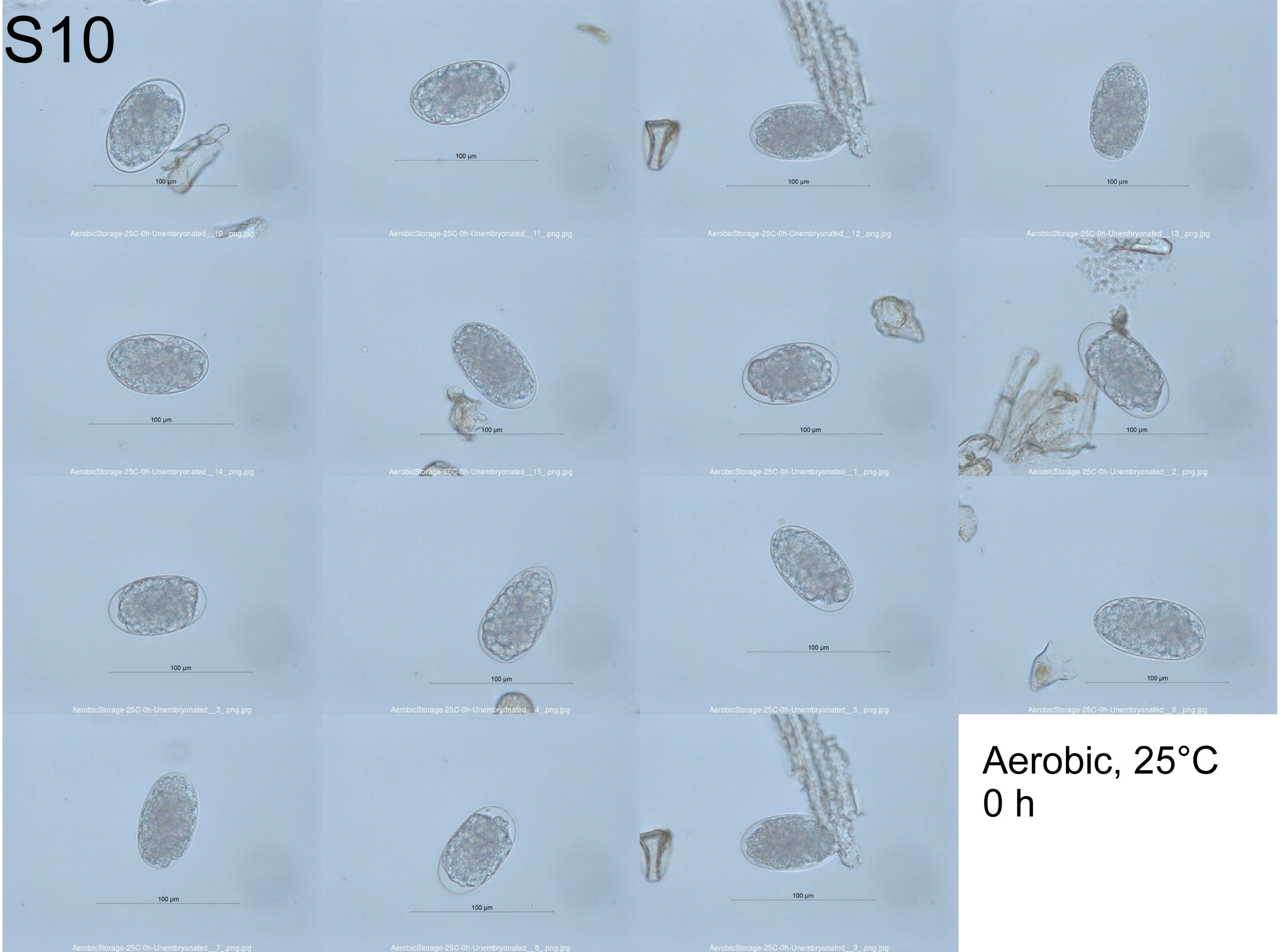

Aerobic, 25°C
0 h

S11

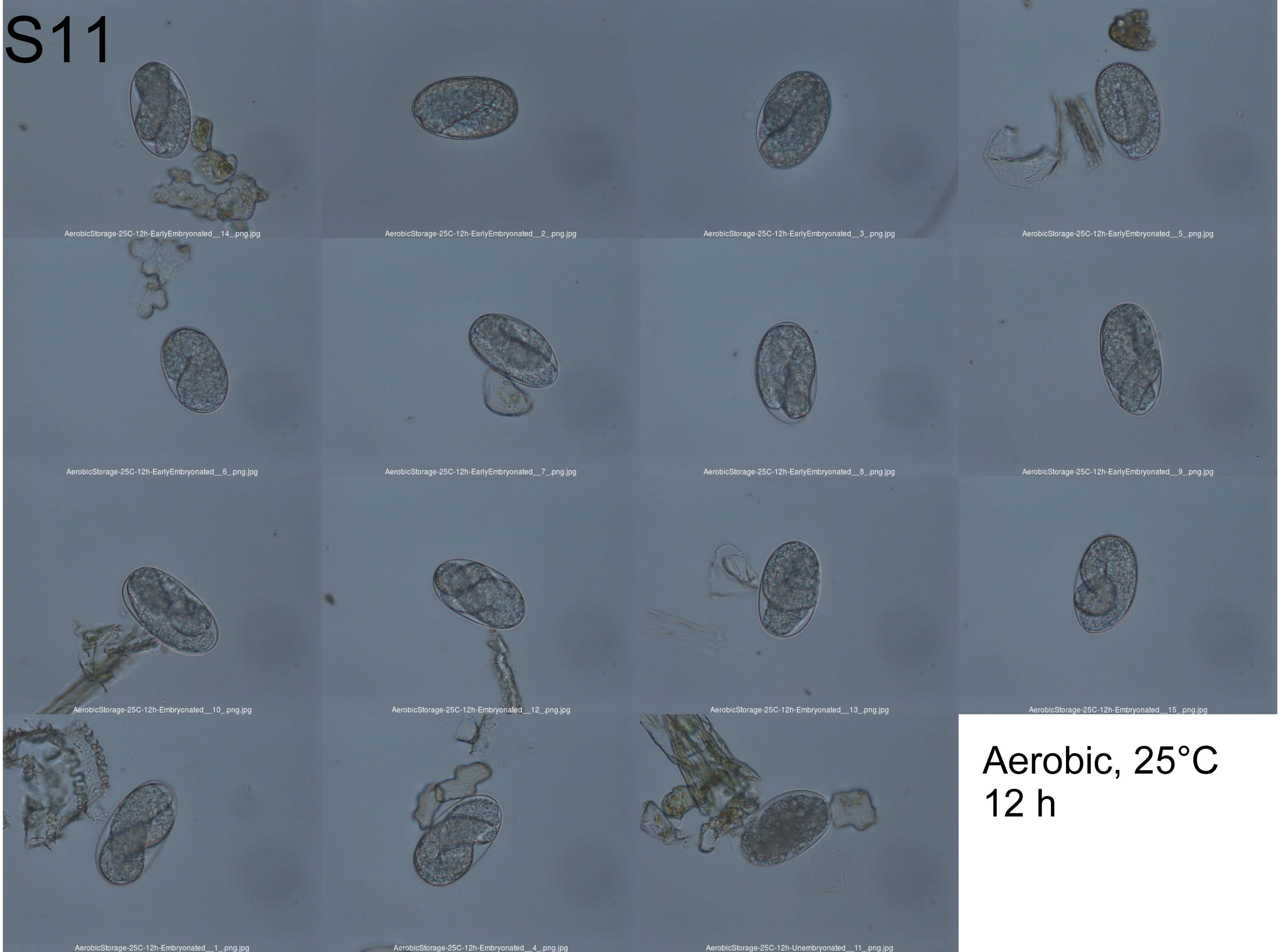

Aerobic, 25°C
12 h

S12

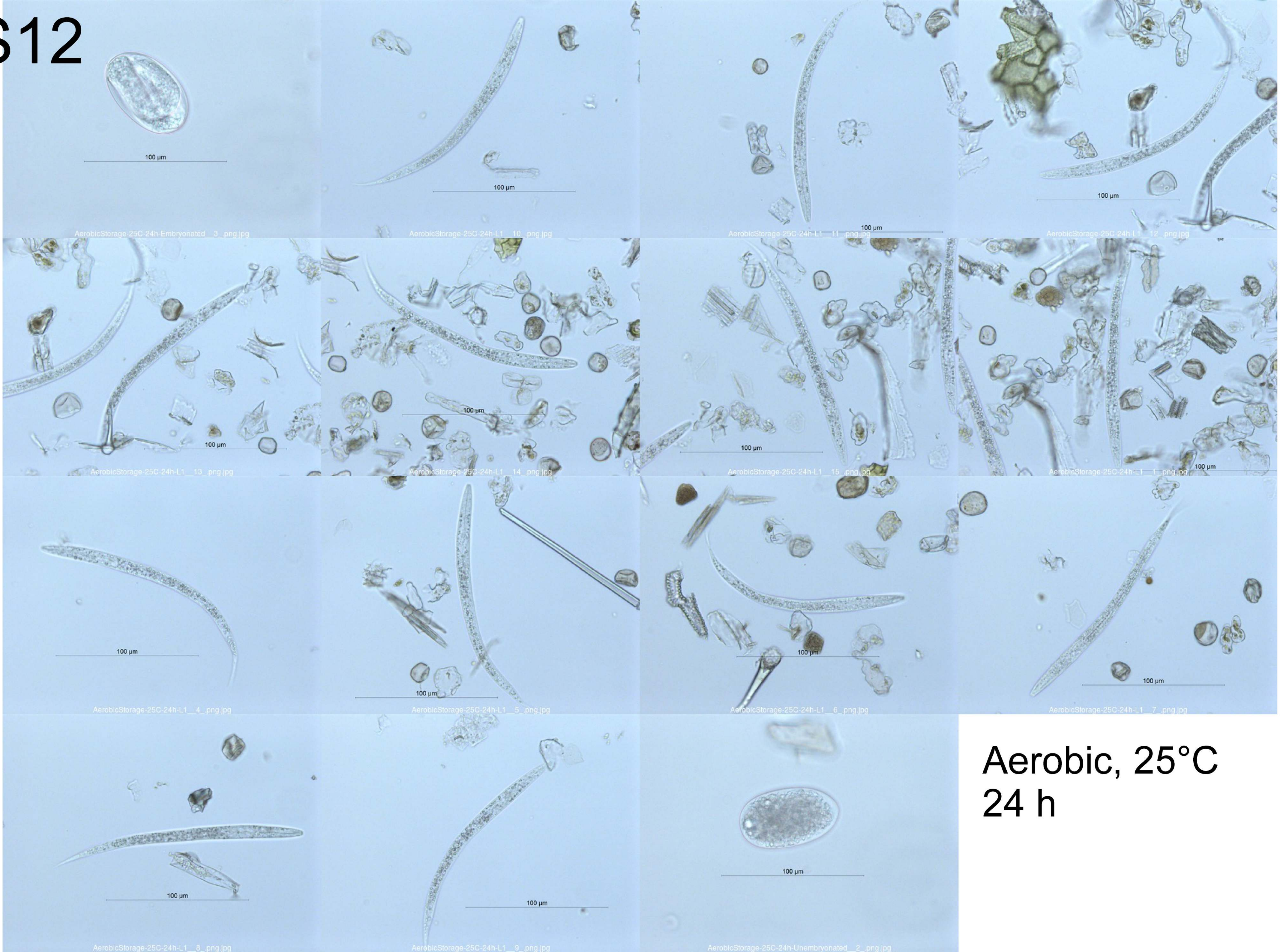
Aerobic, 25°C
24 h
100 µm
AerobicStorage-25C-24h-Embryonated__3_.png.jpg
AerobicStorage-25C-24h-L1__10_.png.jpg
AerobicStorage-25C-24h-L1__11_.png.jpg
AerobicStorage-25C-24h-L1__12_.png.jpg
AerobicStorage-25C-24h-L1__13_.png.jpg
AerobicStorage-25C-24h-L1__14_.png.jpg
AerobicStorage-25C-24h-L1__15_.png.jpg
AerobicStorage-25C-24h-L1__1_.png.jpg
AerobicStorage-25C-24h-L1__4_.png.jpg
AerobicStorage-25C-24h-L1__5_.png.jpg
AerobicStorage-25C-24h-L1__6_.png.jpg
AerobicStorage-25C-24h-L1__7_.png.jpg
AerobicStorage-25C-24h-L1__8_.png.jpg
AerobicStorage-25C-24h-L1__9_.png.jpg
AerobicStorage-25C-24h-Unembryonated__2_.png.jpg

S13

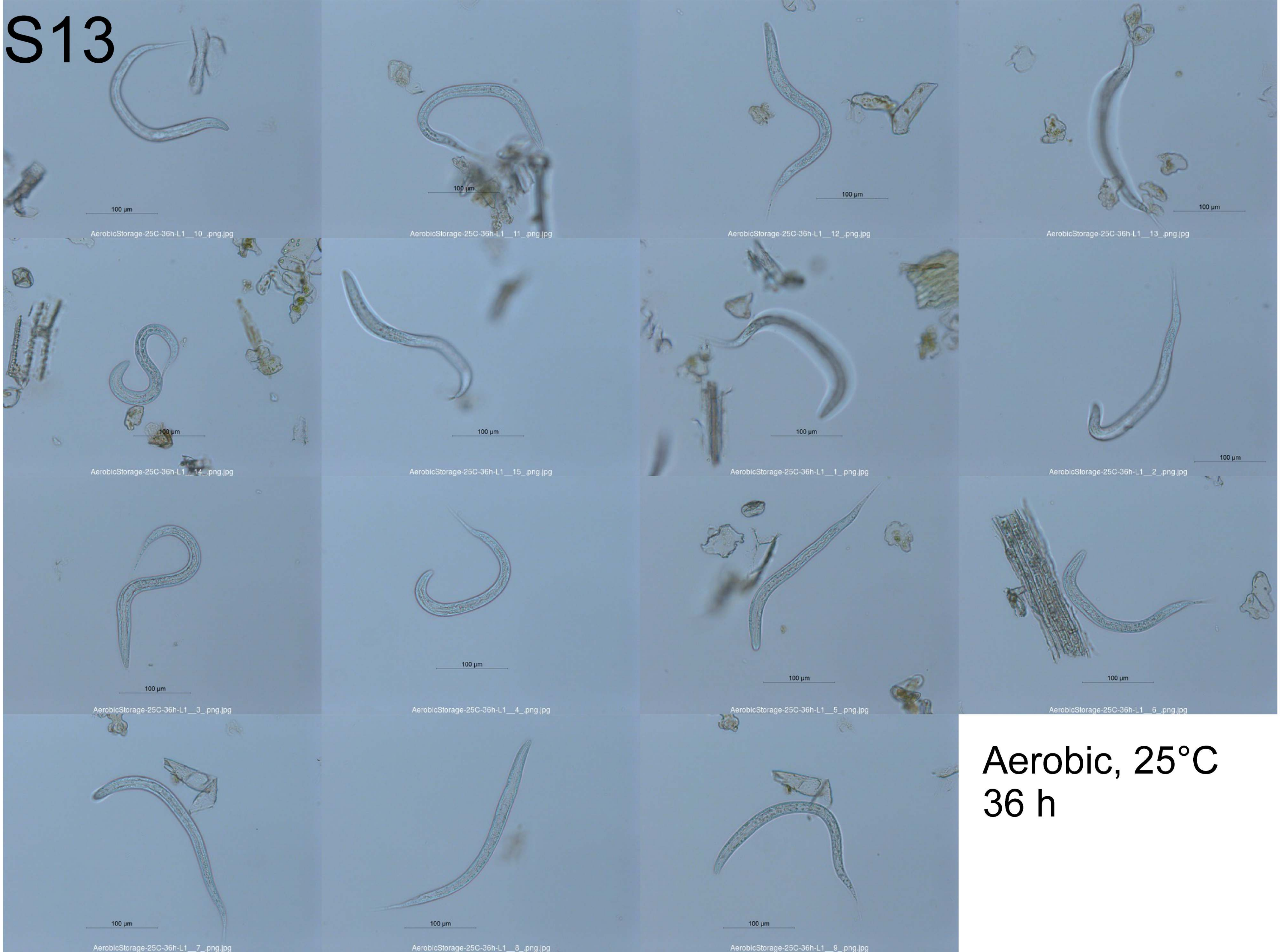

Aerobic, 25°C
36 h

S14

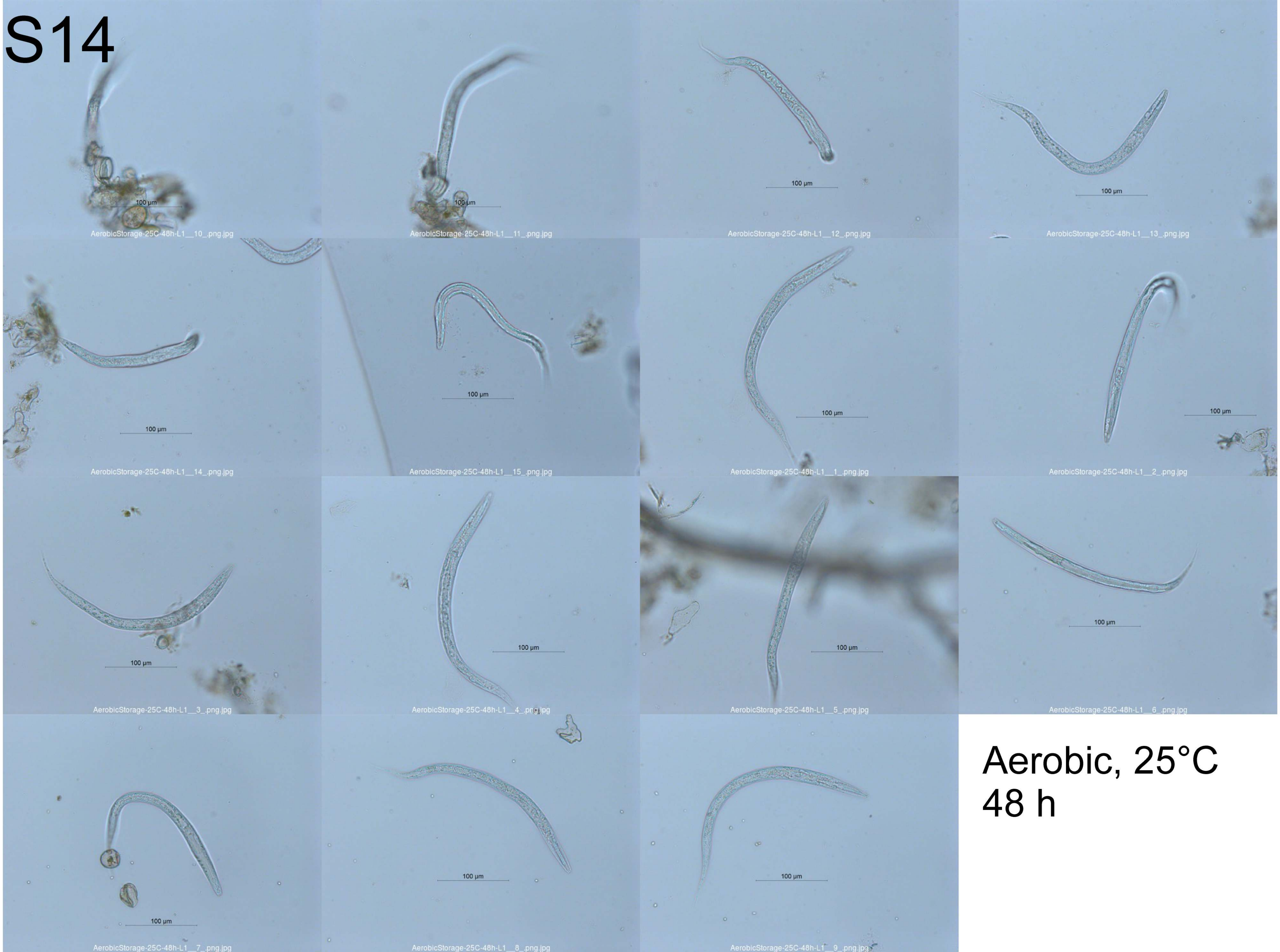

Aerobic, 25°C
48 h

S15

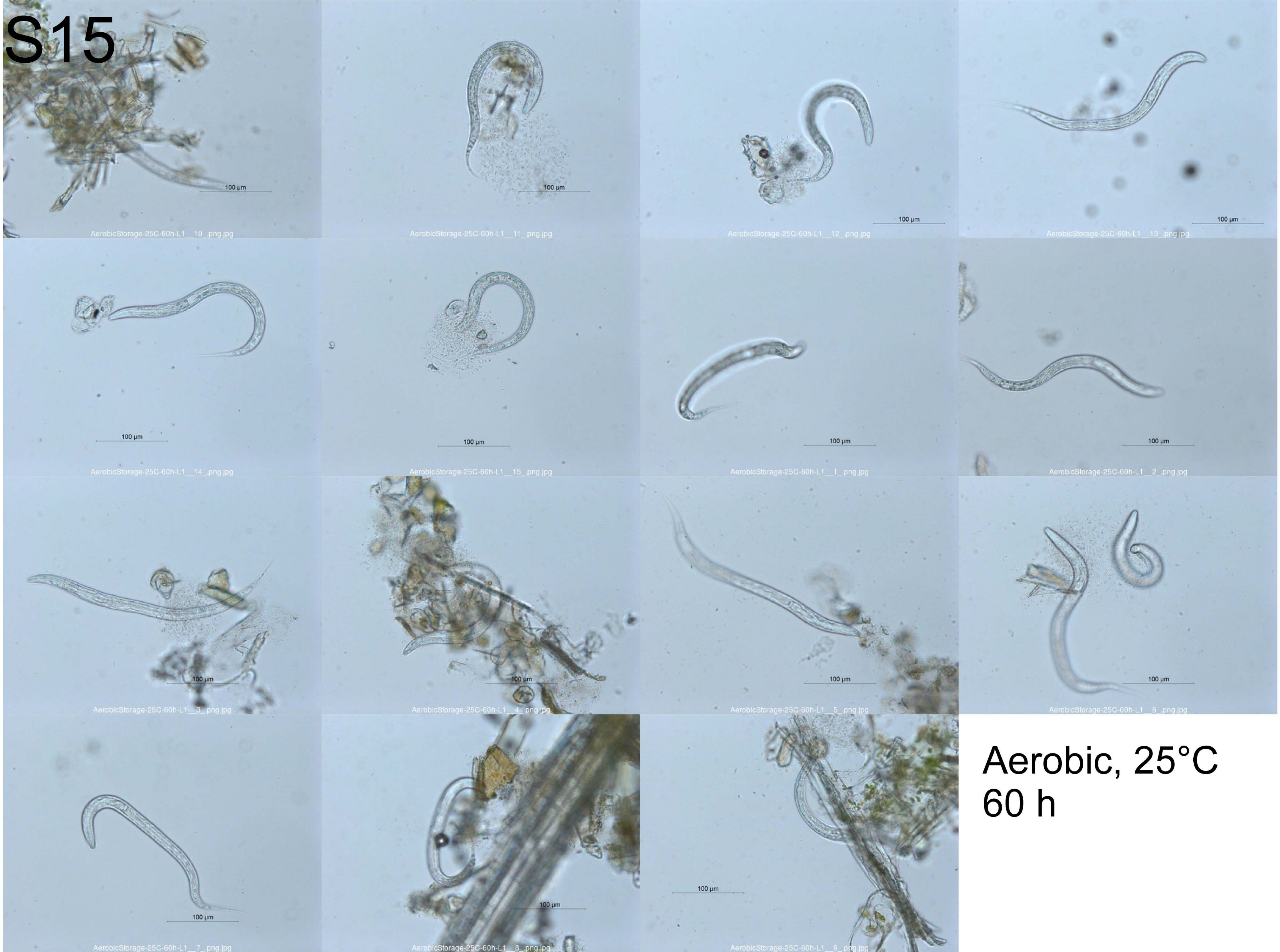

Aerobic, 25°C
60 h

S16

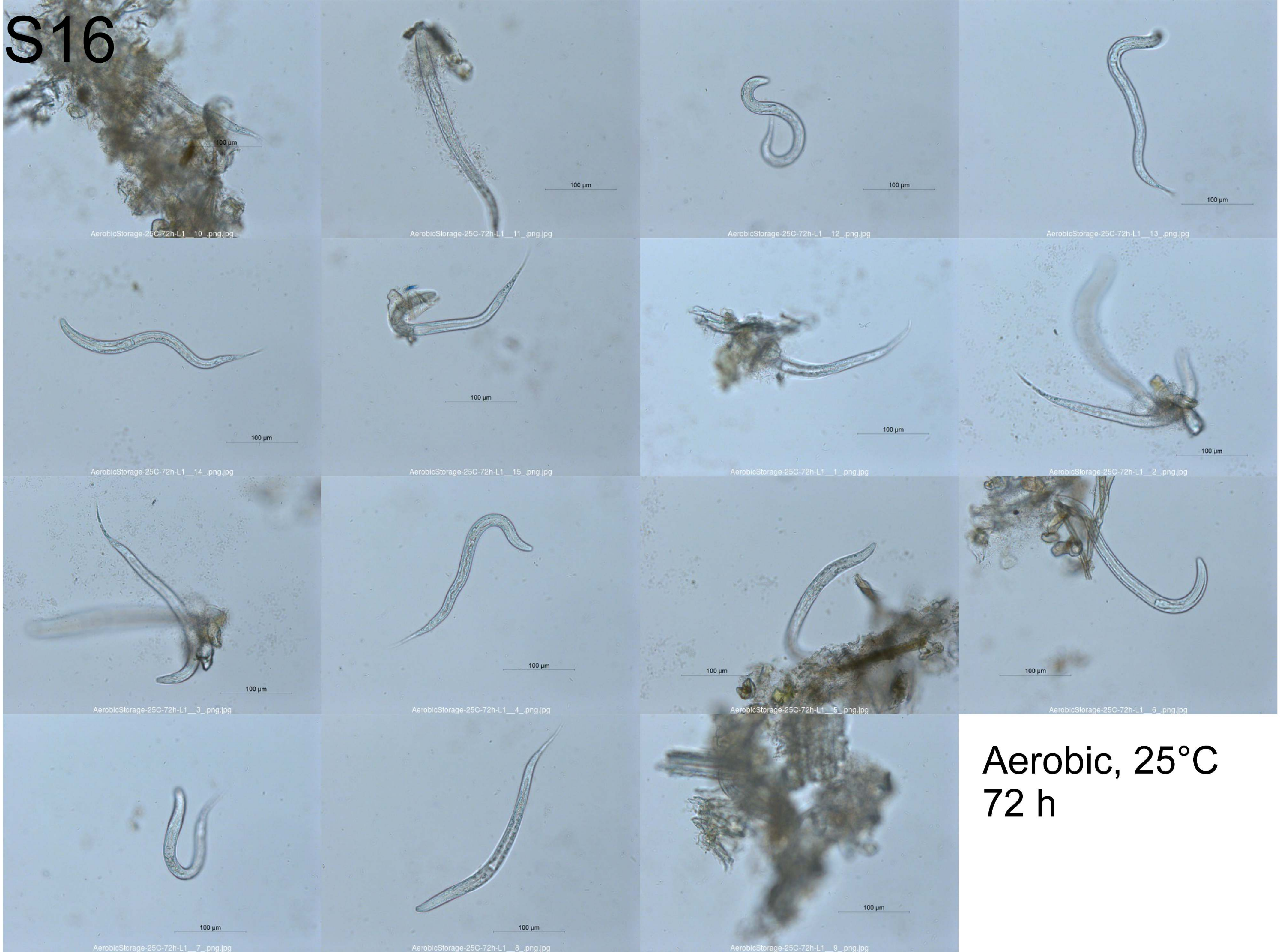

Aerobic, 25°C
72 h

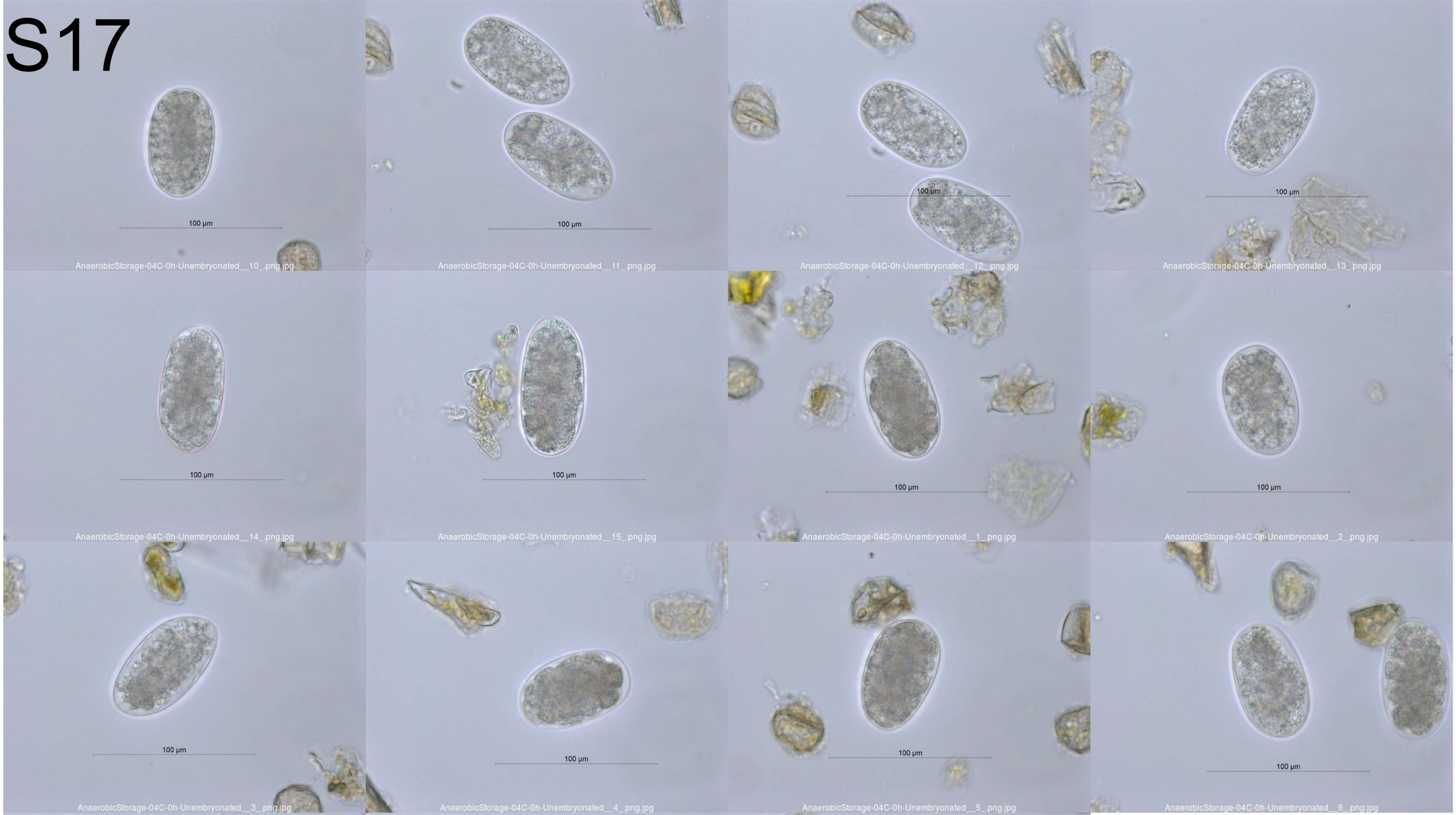

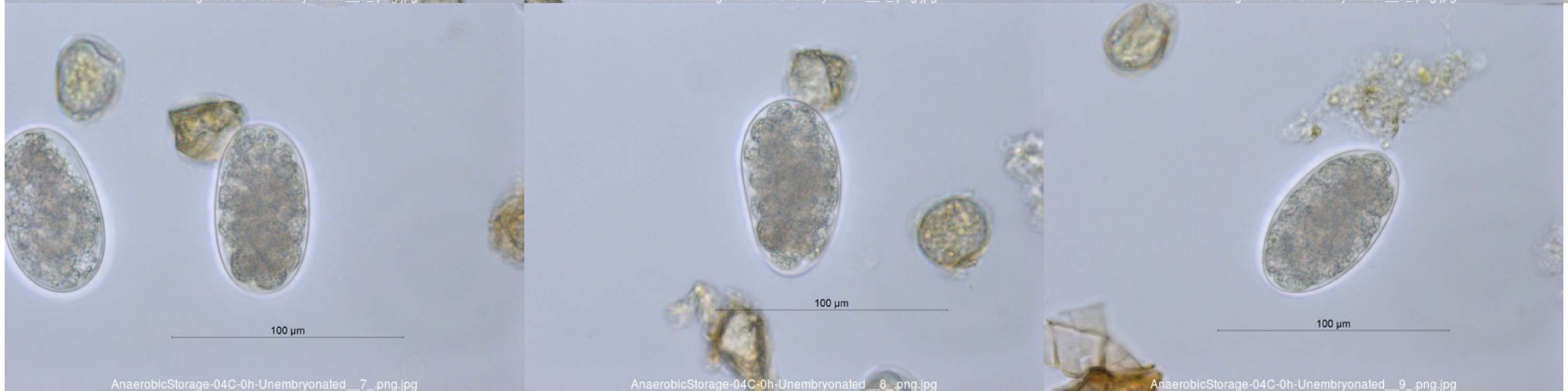

Anaerobic, 4°C
0 h

S18

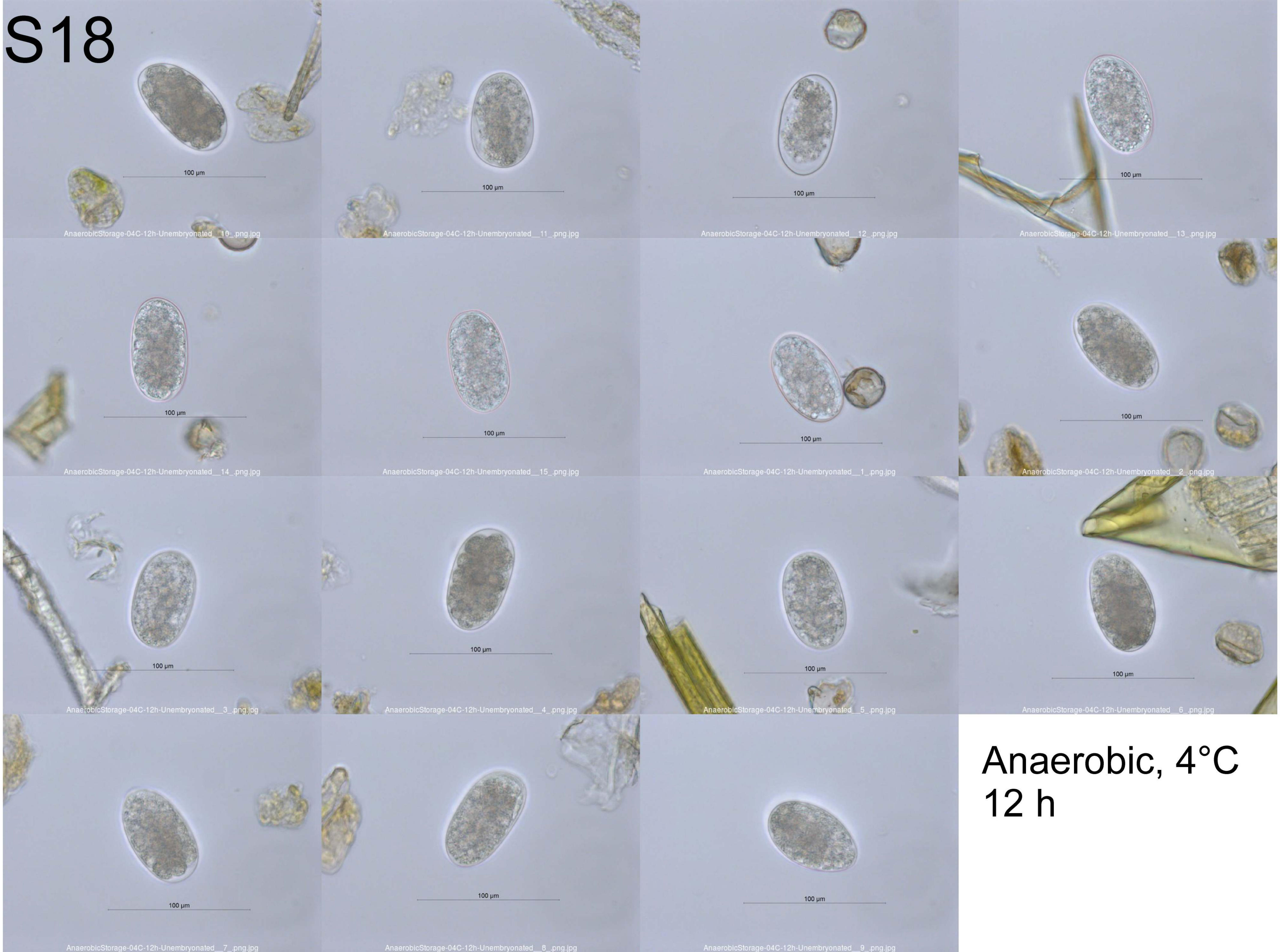

Anaerobic, 4°C
12 h

S19

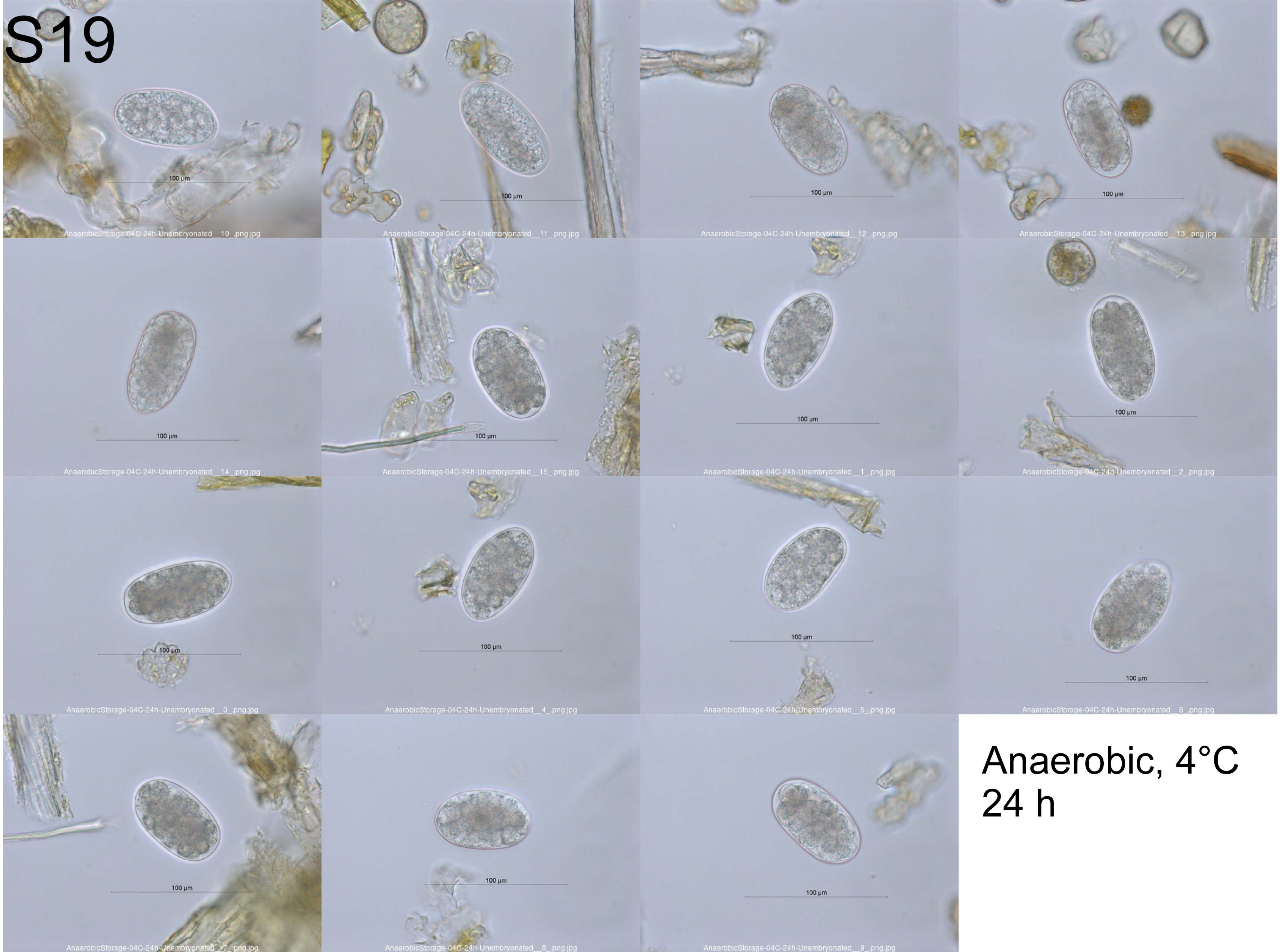

Anaerobic, 4°C
24 h

S20

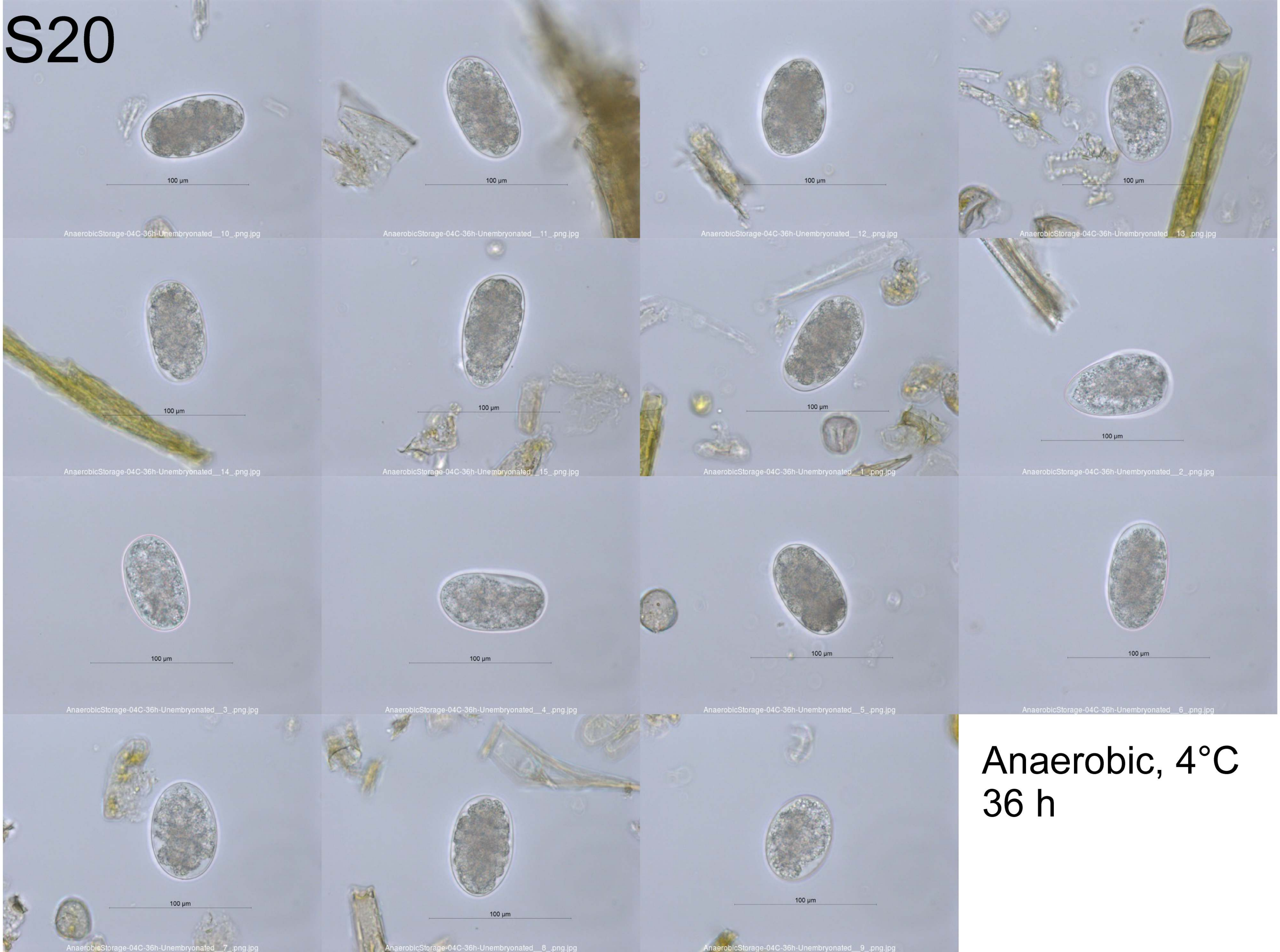

Anaerobic, 4°C
36 h

S21

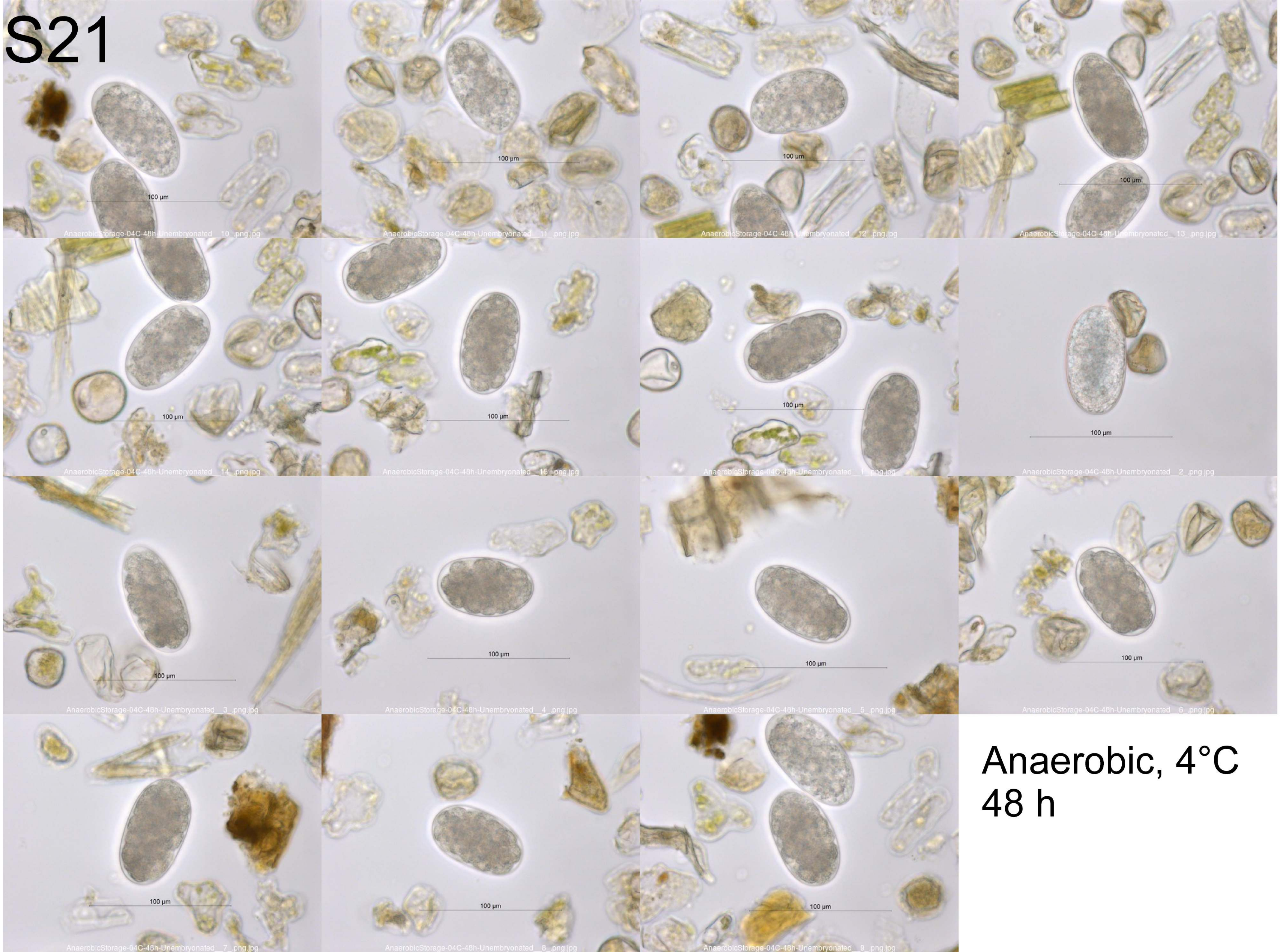

Anaerobic, 4°C
48 h

S22

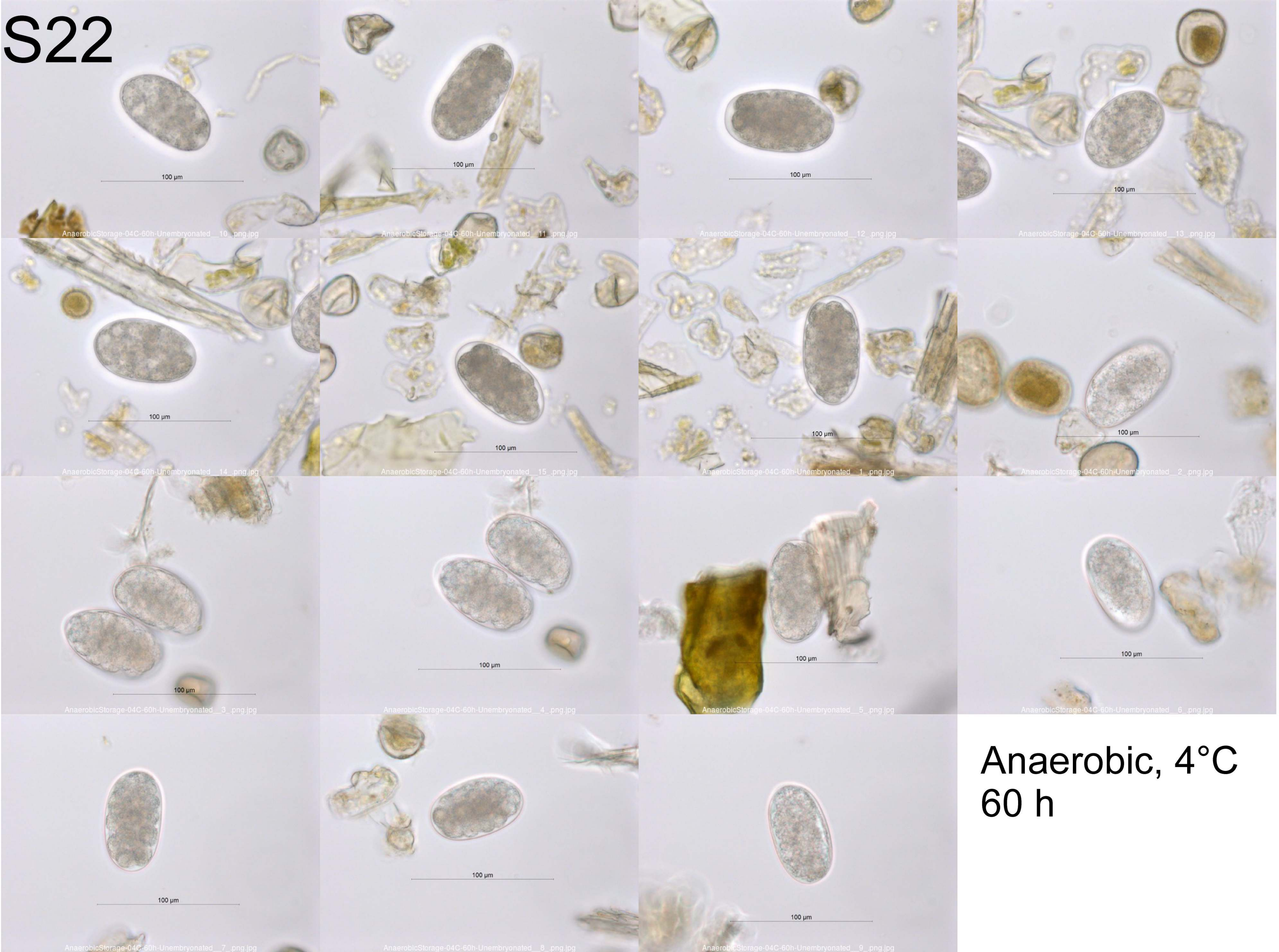

Anaerobic, 4°C
60 h

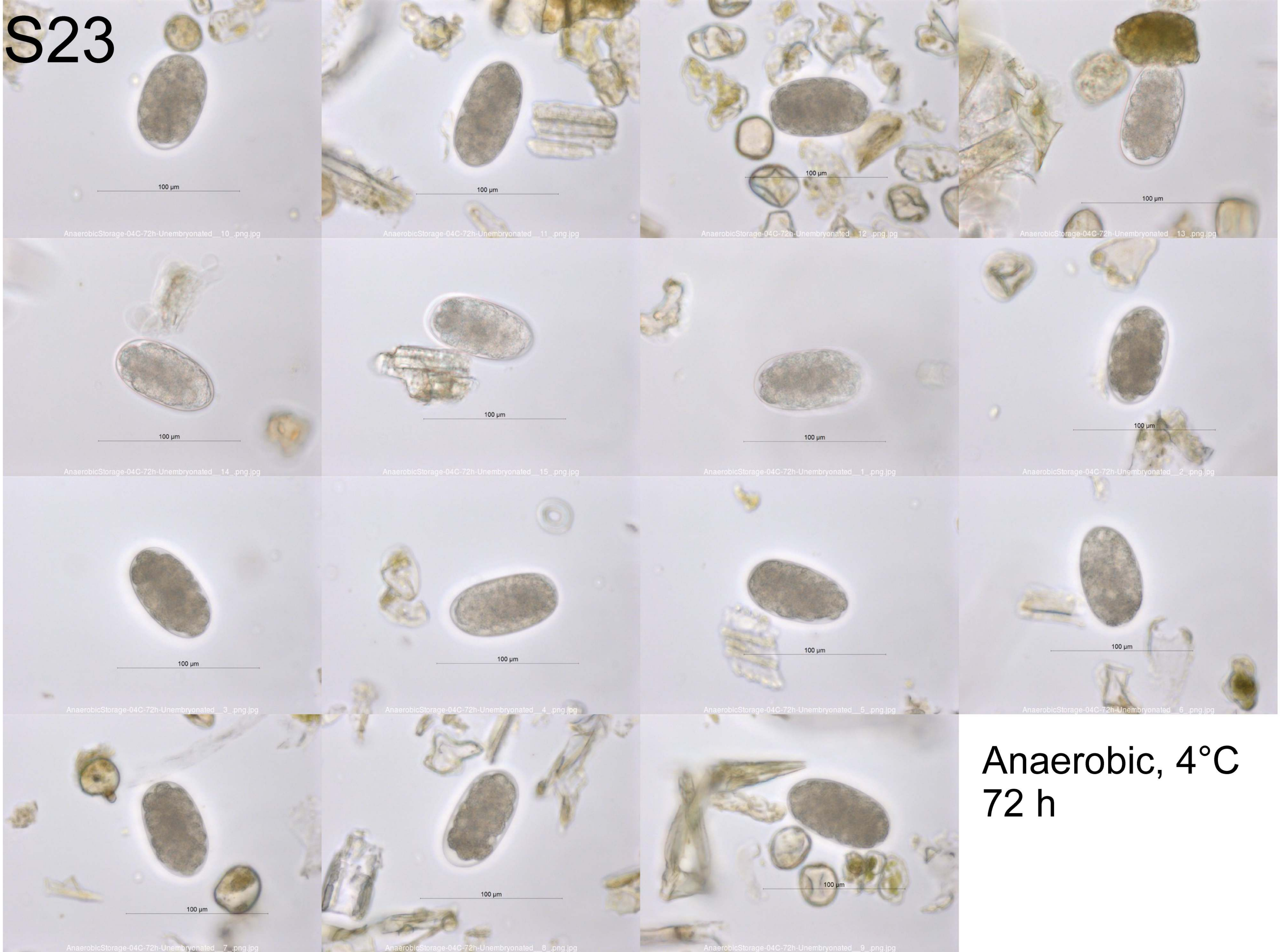

Anaerobic, 4°C
72 h

S24

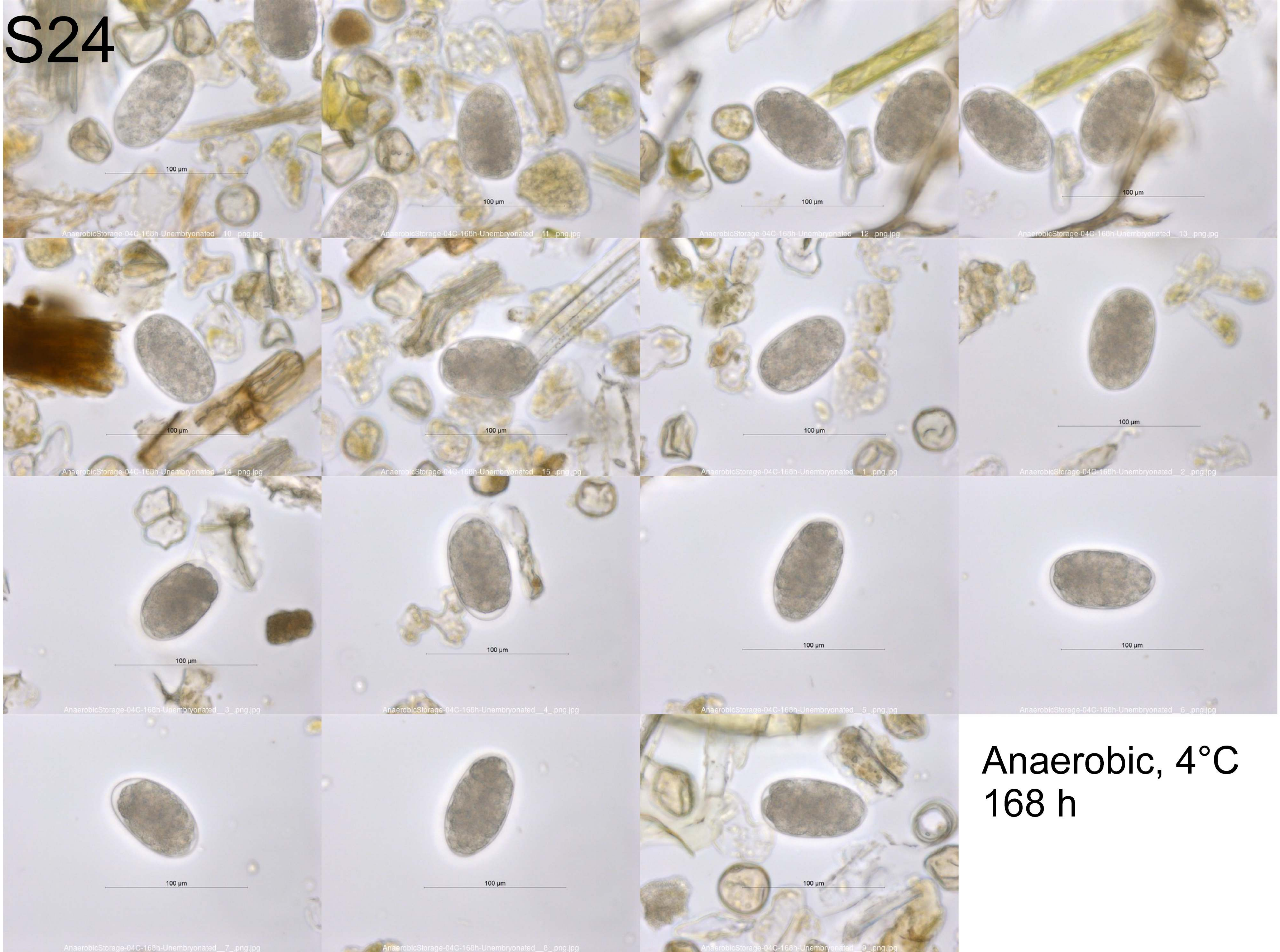

Anaerobic, 4°C
168 h

S25

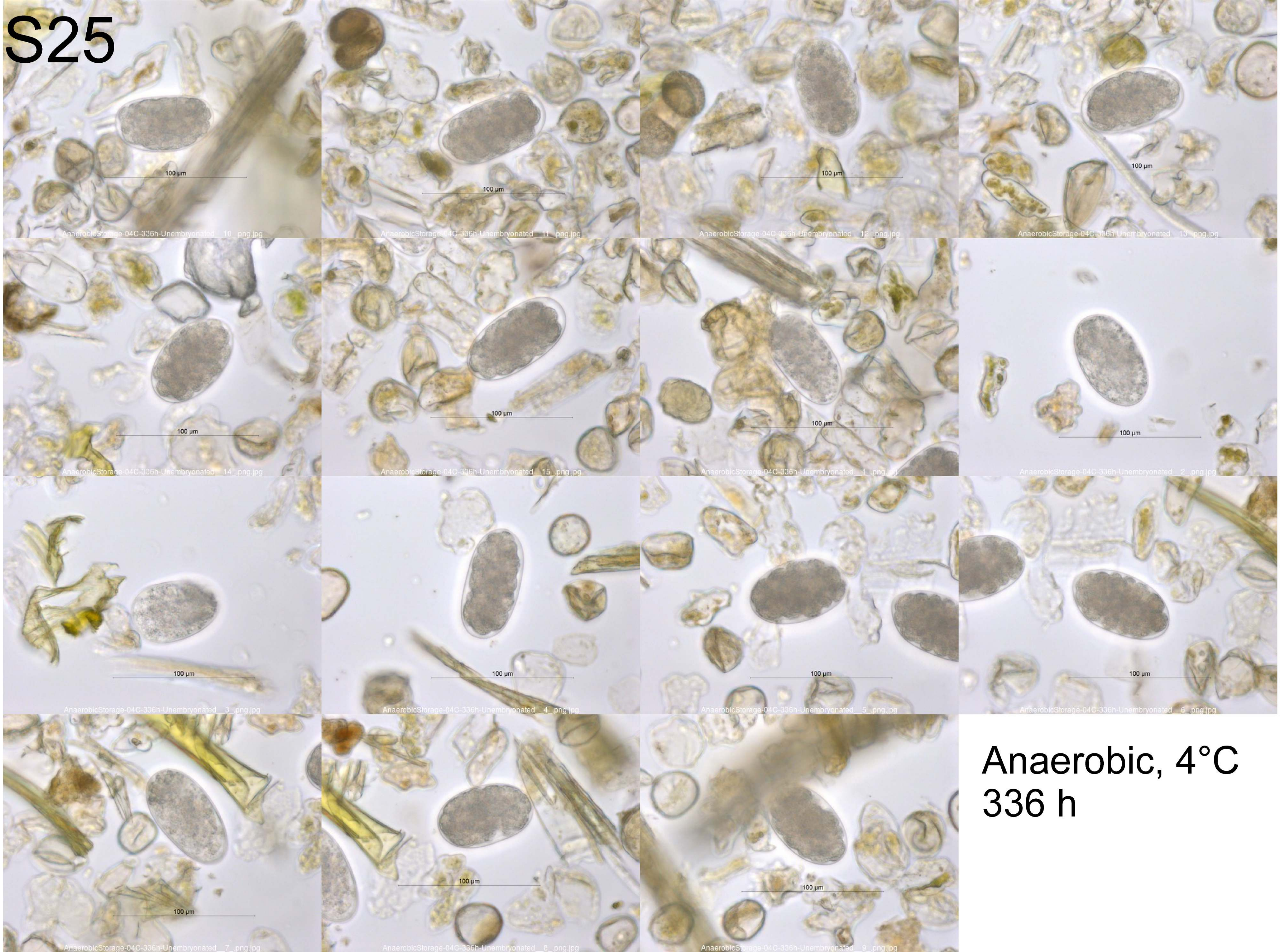

Anaerobic, 4°C
336 h

S26

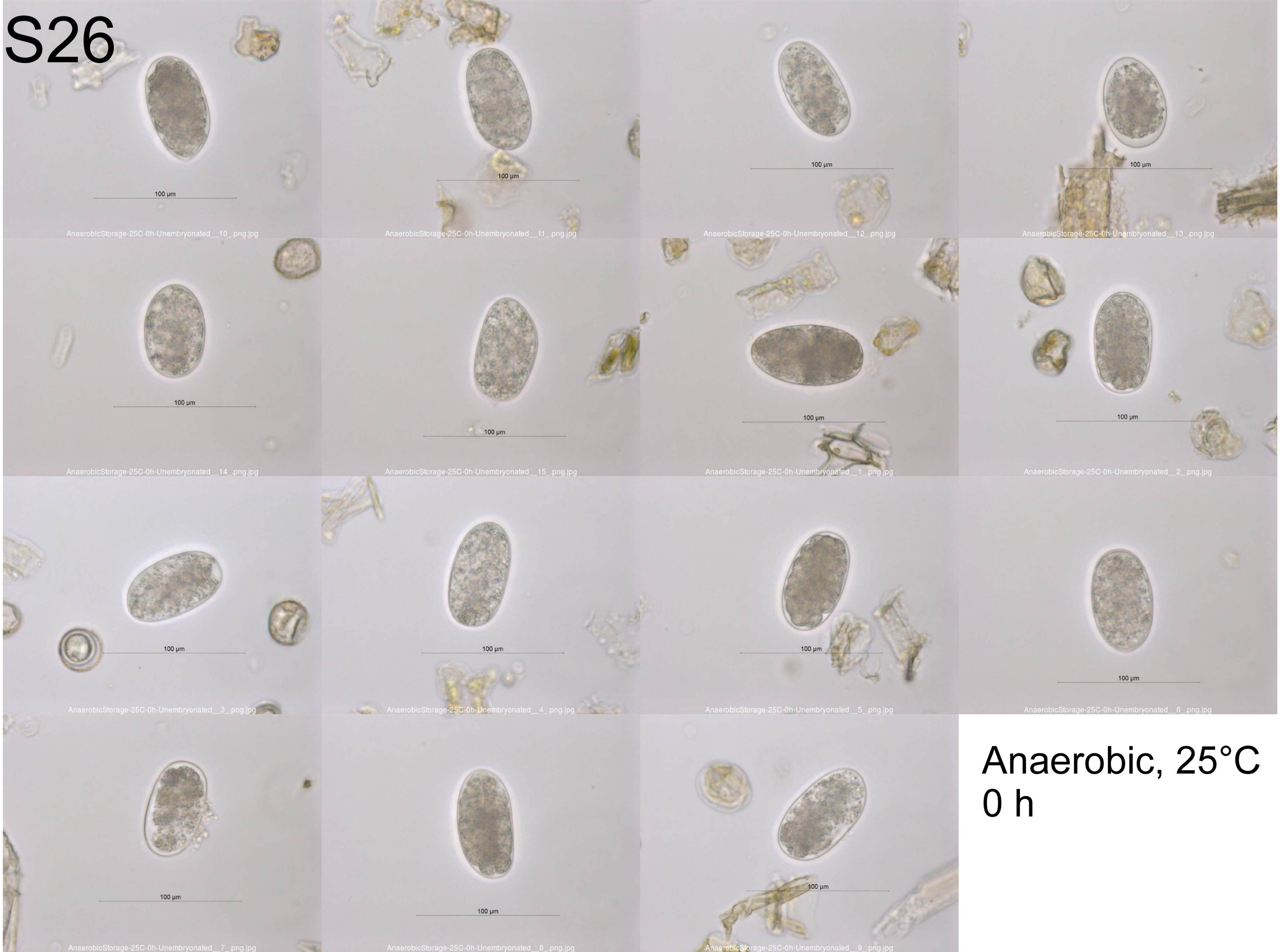

Anaerobic, 25°C
0 h

S27

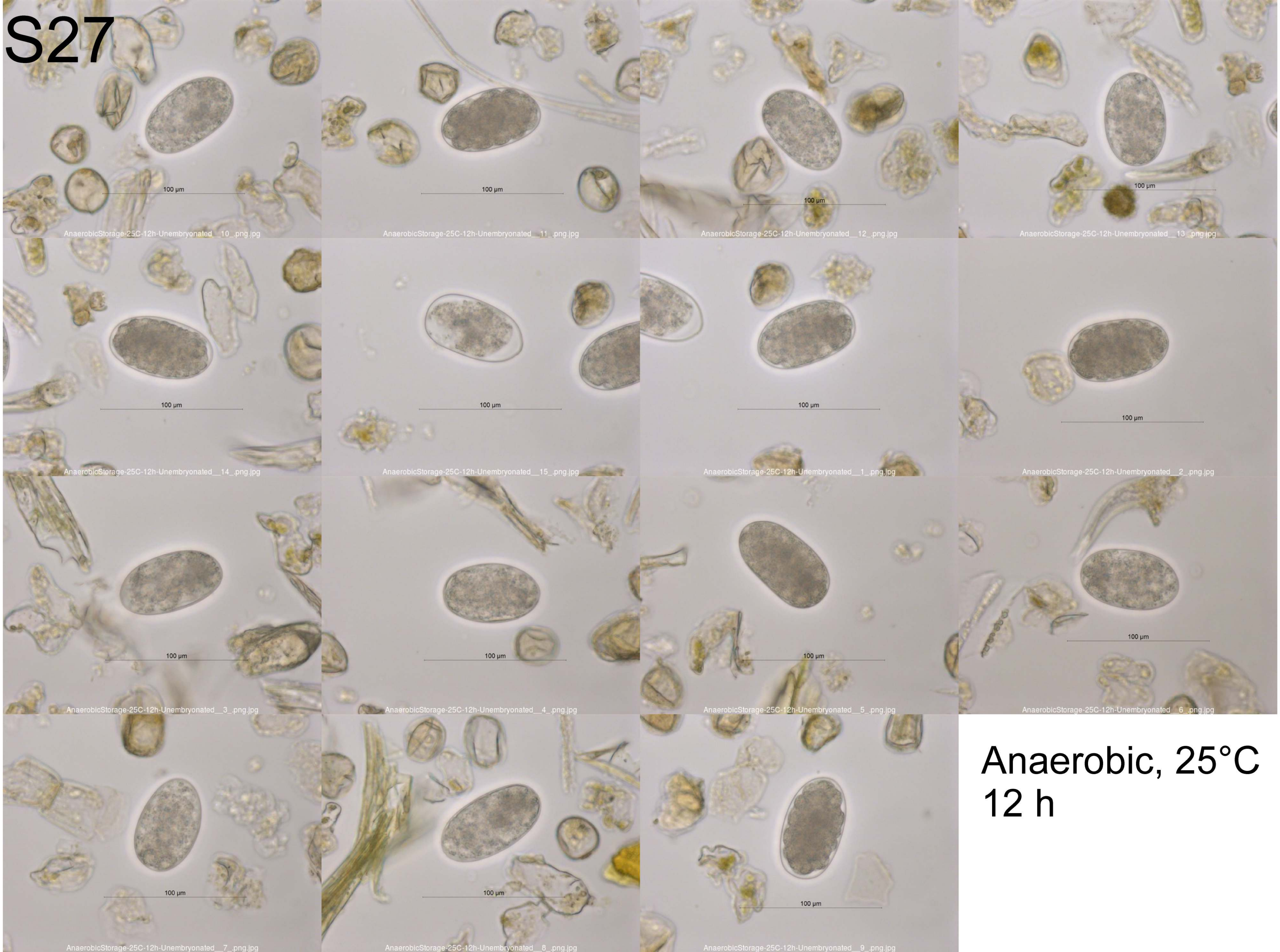

Anaerobic, 25°C
12 h

S28

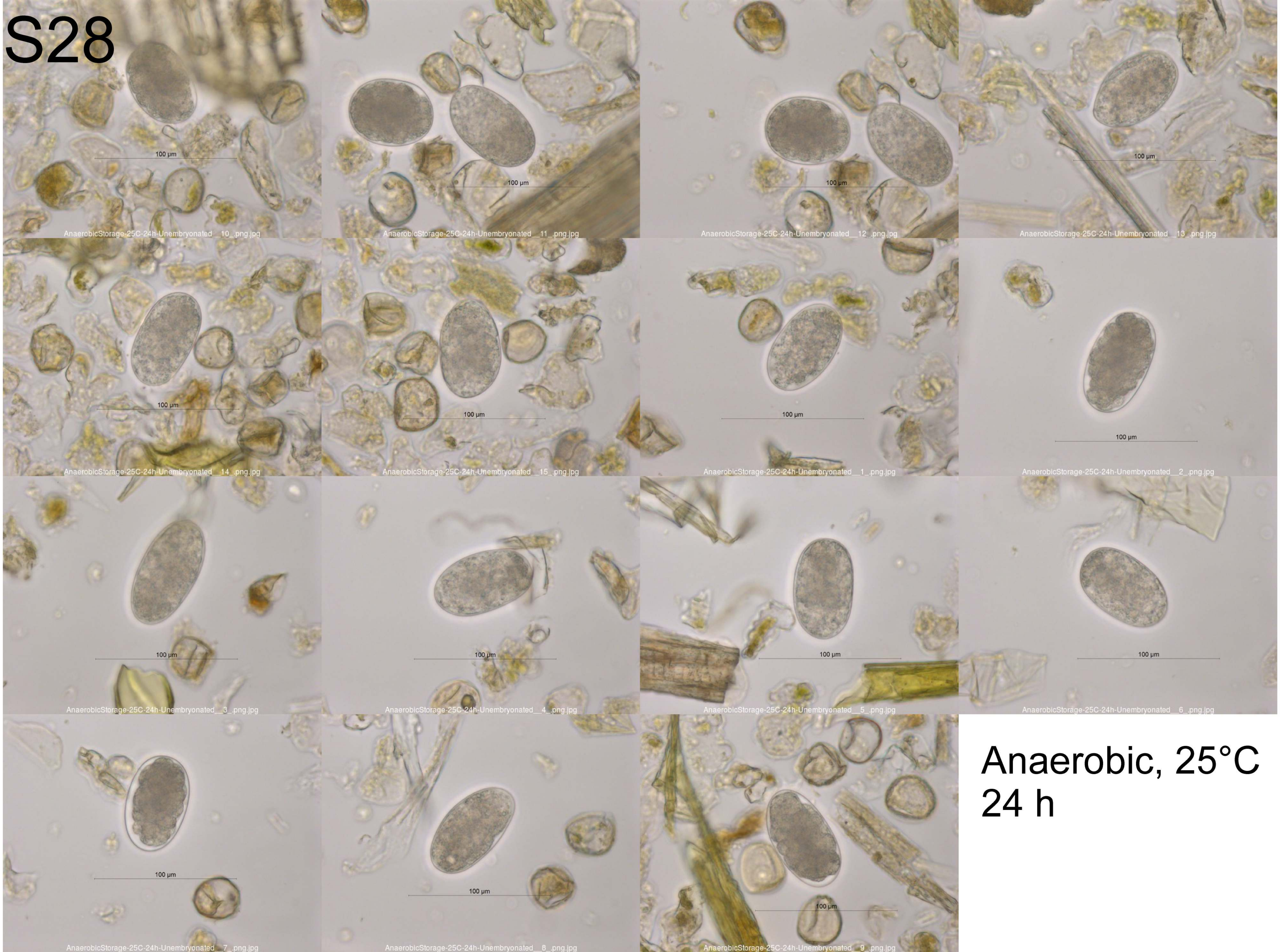

Anaerobic, 25°C
24 h

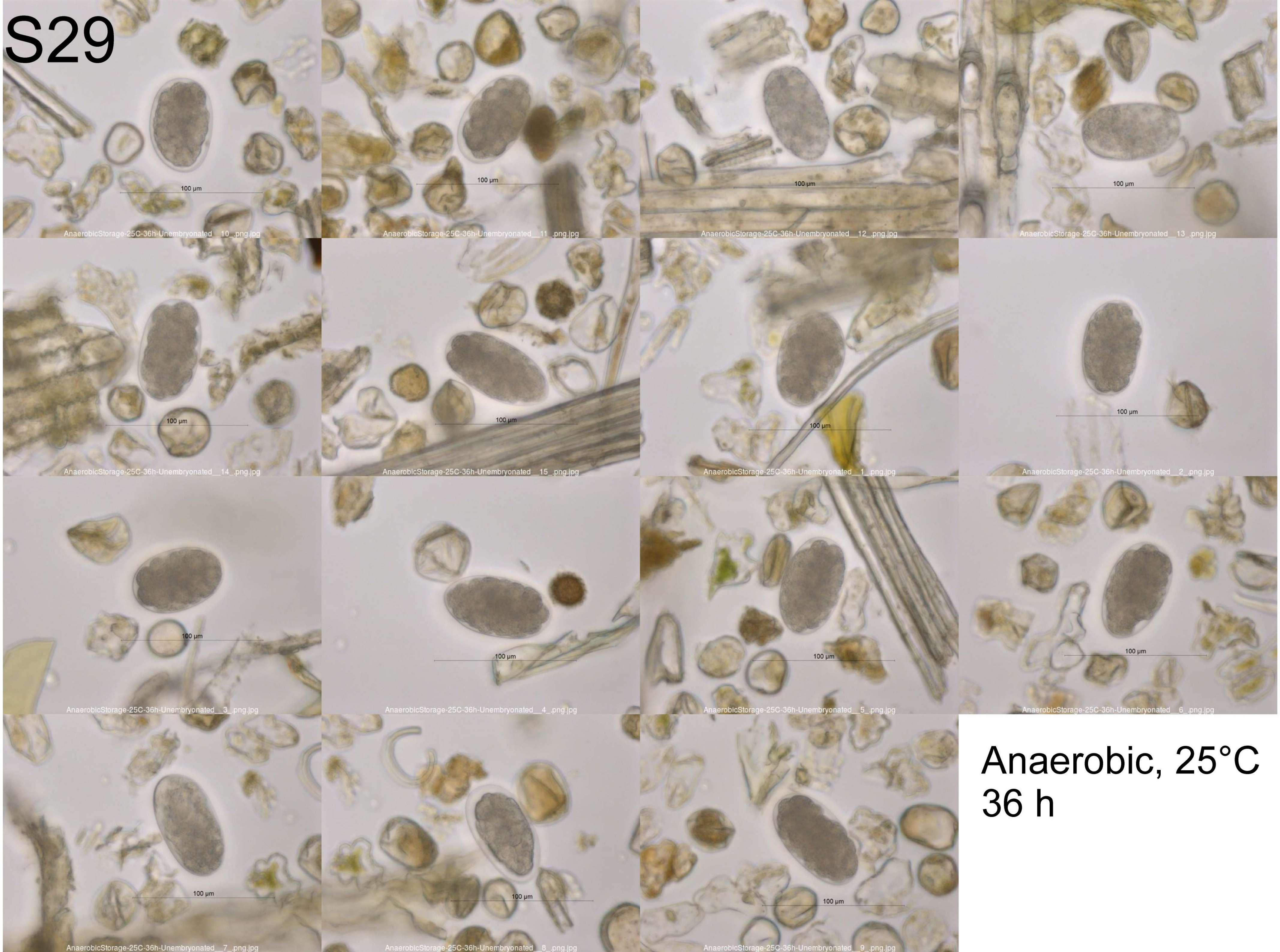

Anaerobic, 25°C
36 h

S30

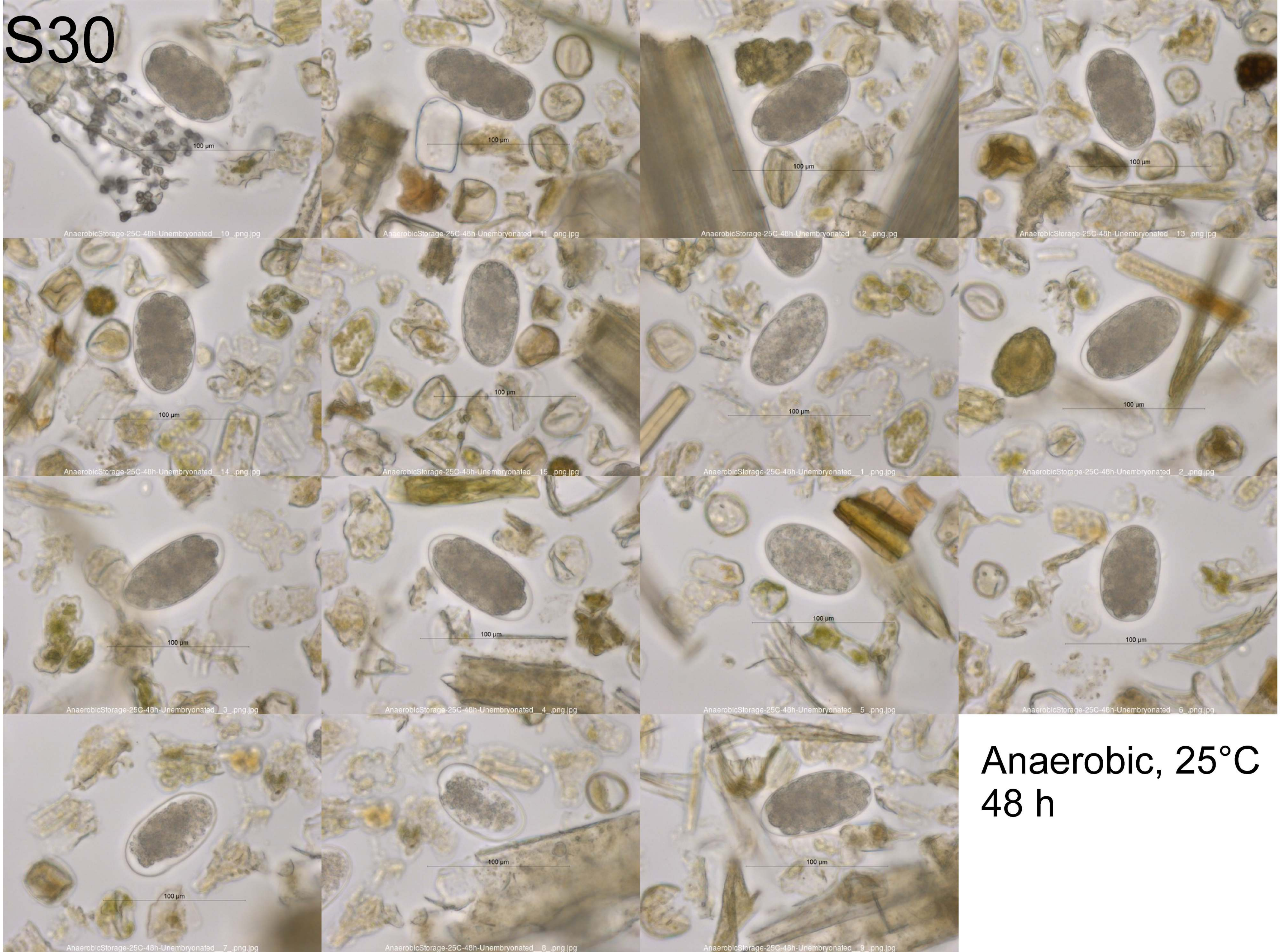

Anaerobic, 25°C
48 h

S31

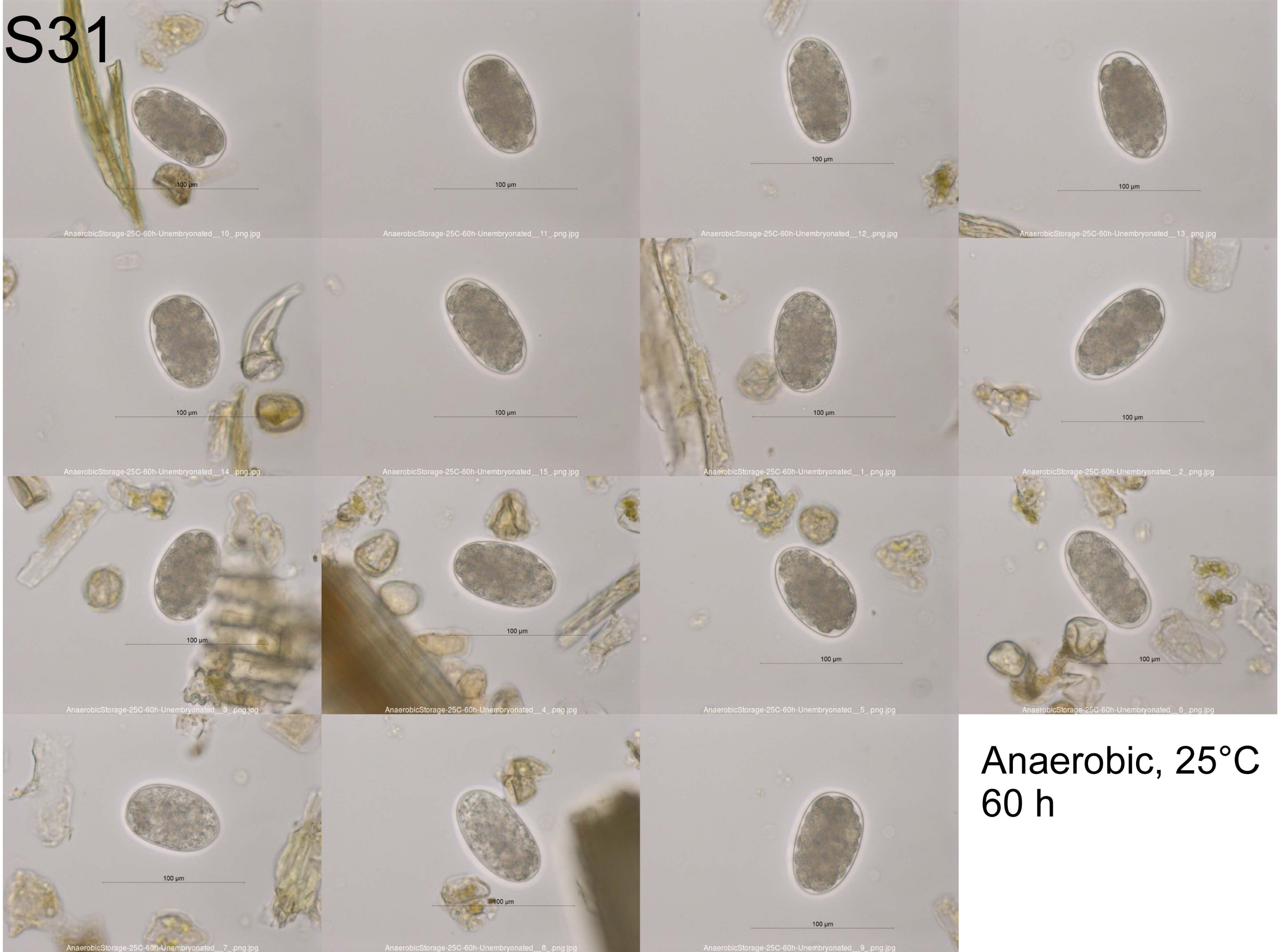

Anaerobic, 25°C
60 h

S32

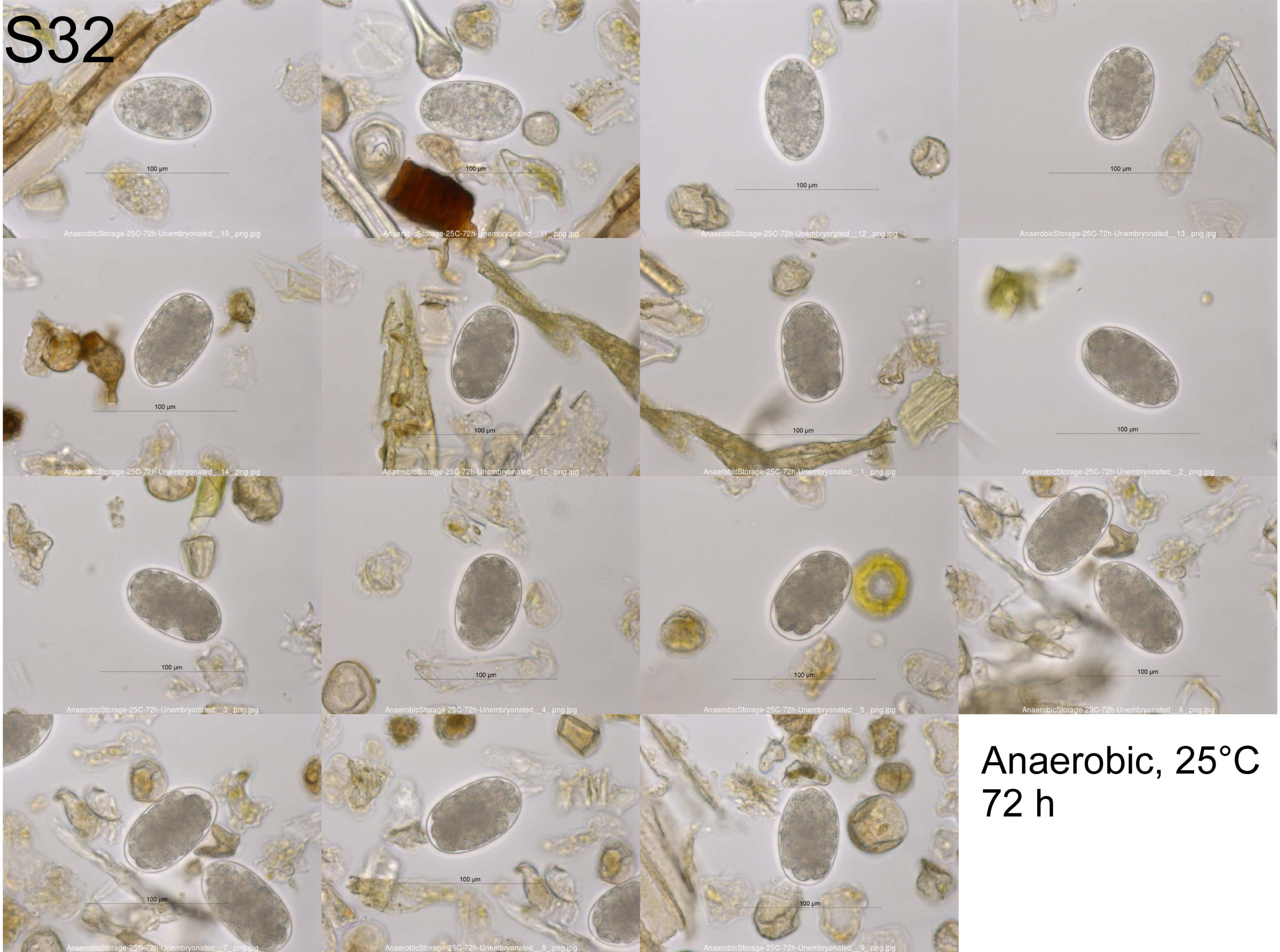

Anaerobic, 25°C
72 h

S33

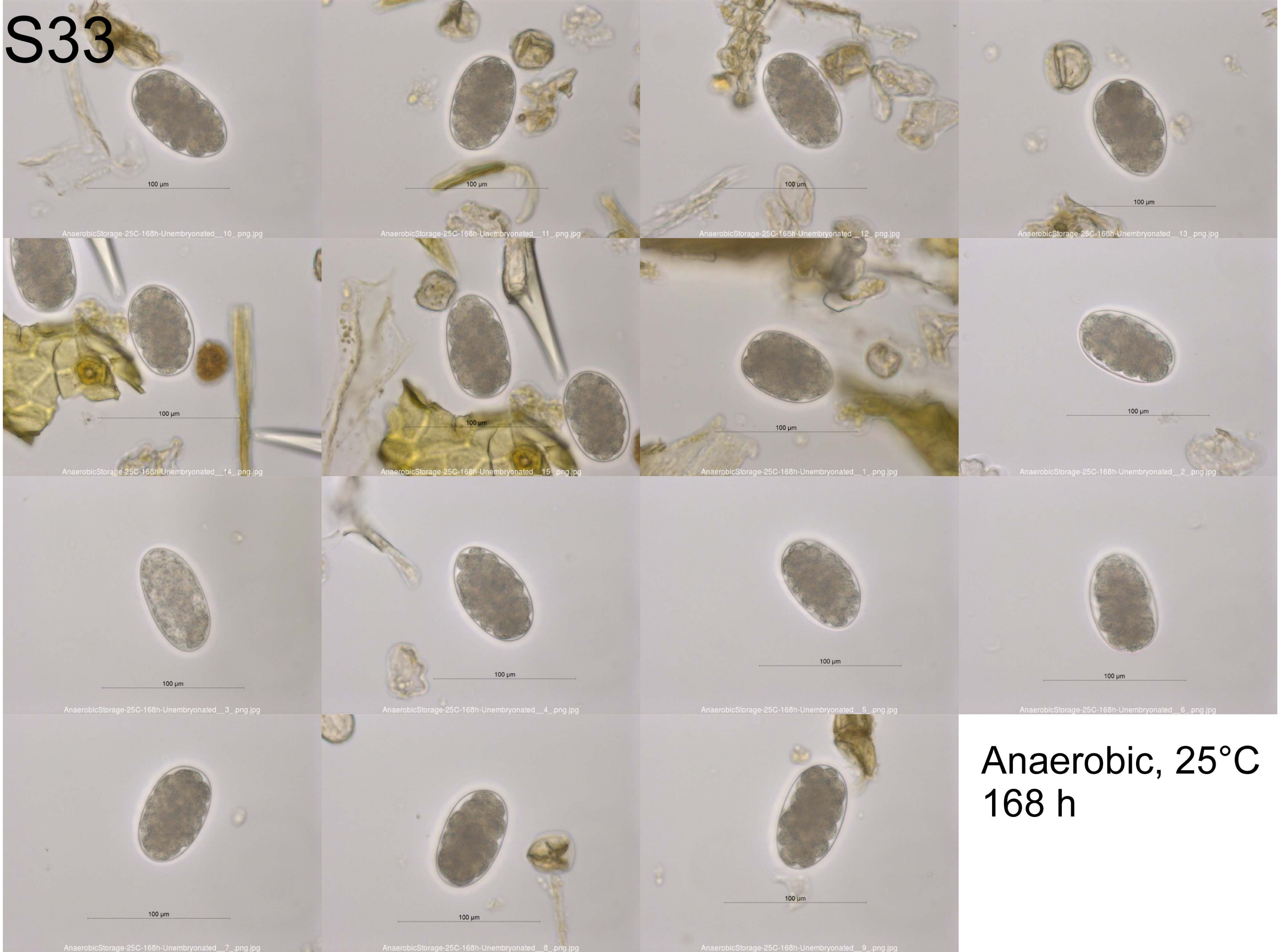

Anaerobic, 25°C
168 h

S34

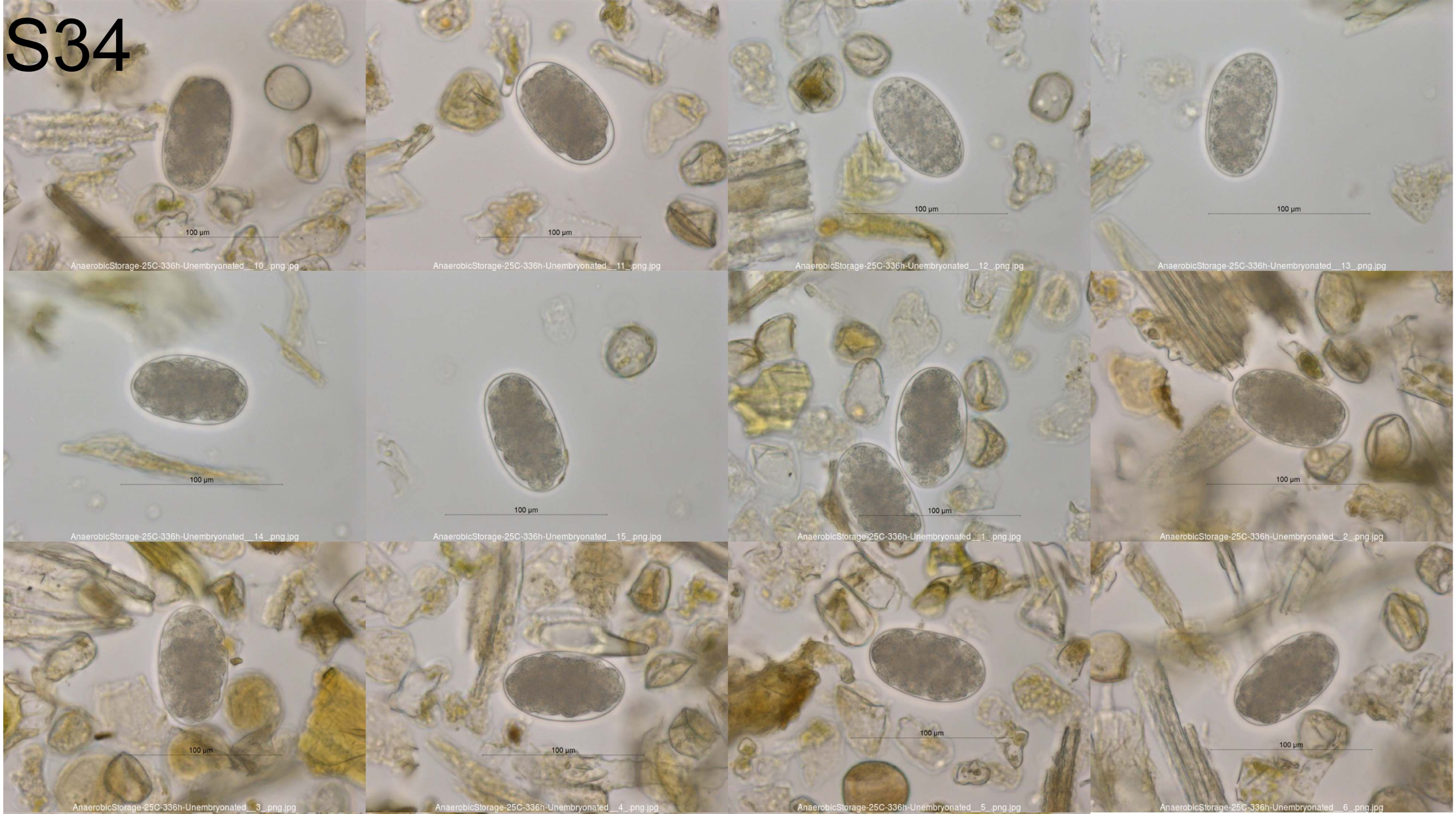

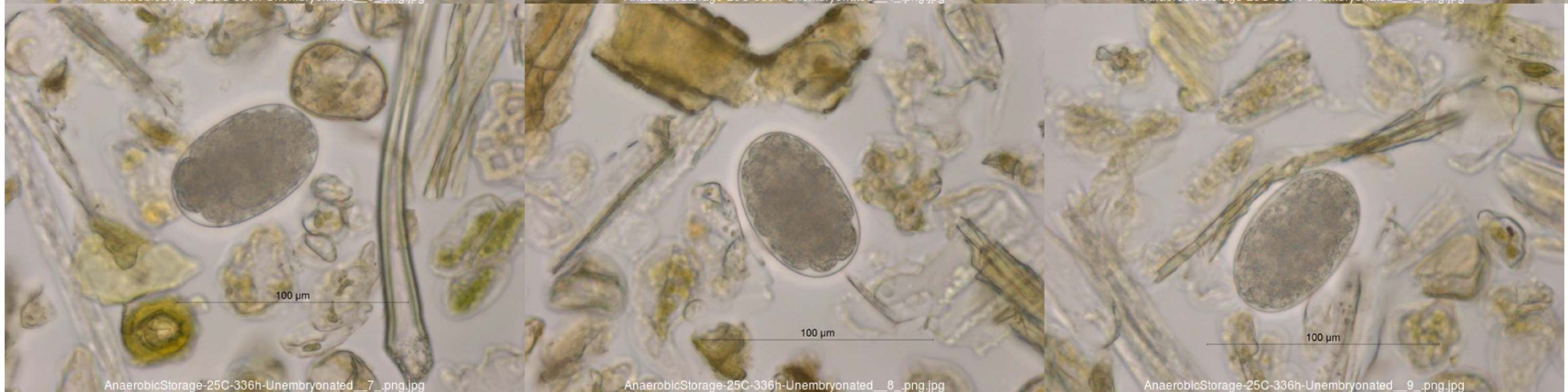

Anaerobic, 25°C
336 h
