# Supplementary material for: The level of embryonation influences detection of Ostertagia ostertagi eggs by semi-quantitative PCR
Source: Parasit Vectors. 2016 Jun 29;9:368. doi: 10.1186/s13071-016-1657-4 (PMC4928311; doi:10.1186/s13071-016-1657-4)
Supplement: Additional file 2: — Alignment of 49 ITS2 sequences from Ostertagia ostertagi obtained from BLAST on the O. ostertagi ITS2 sequence (GenBank AB245021.2|: 1,036–1,126 bp) targeted by the qPCR developed by Höglund et al. [15] and used in this study. Primers and probe are aligned in the bottom. Asterisks indicate the complete nucleotide conservation throughout all sequences; 15 sequences (31 %) contained single nucleotide polymorphisms (SNPs) in the forward primer region. (DOC 35 kb) [file 13071_2016_1657_MOESM2_ESM.doc]

CLUSTAL Omega (1.2.1) multiple sequence alignment of ITS2 sequences from *Ostertagia ostertagi*

gi|163247540|dbj|AB245021.2|:1036-1126 ATGAAACTACTACAGTGTGGCTAGTTCATAACACTGTTTGTCGAATGGTATTTATTACTT 60

gi|507147019|gb|KC998718.1|:154-244 ATGAAACTACTACAGTGTGGCTAGTTCATAACACTGTTTGTCGAATGGTATTTATTACTT 60

gi|357429009|dbj|AB682689.1|:914-1004 ATGAAACTACTACAGTGTGGCTAGTTCATAACACTGTTTGTCGAATGGTATTTATTACTT 60

gi|357429008|dbj|AB682688.1|:914-1004 ATGAAACTACTACAGTGTGGCTAGTTCATAACACTGTTTGTCGAATGGTATTTATTACTT 60

gi|163247543|dbj|AB245024.2|:1036-1126 ATGAAACTACTACAGTGTGGCTAGTTCATAACACTGTTTGTCGAATGGTATTTATTACTT 60

gi|163247542|dbj|AB245023.2|:1036-1126 ATGAAACTACTACAGTGTGGCTAGTTCATAACACTGTTTGTCGAATGGTATTTATTACTT 60

gi|163247539|dbj|AB245020.2|:1036-1126 ATGAAACTACTACAGTGTGGCTAGTTCATAACACTGTTTGTCGAATGGTATTTATTACTT 60

gi|163247537|dbj|AB245018.2|:1036-1126 ATGAAACTACTACAGTGTGGCTAGTTCATAACACTGTTTGTCGAATGGTATTTATTACTT 60

gi|163247536|dbj|AB245017.2|:1036-1126 ATGAAACTACTACAGTGTGGCTAGTTCATAACACTGTTTGTCGAATGGTATTTATTACTT 60

gi|163247535|dbj|AB245016.2|:1036-1126 ATGAAACTACTACAGTGTGGCTAGTTCATAACACTGTTTGTCGAATGGTATTTATTACTT 60

gi|163247534|dbj|AB245015.2|:1036-1126 ATGAAACTACTACAGTGTGGCTAGTTCATAACACTGTTTGTCGAATGGTATTTATTACTT 60

gi|163247533|dbj|AB245014.2|:1036-1126 ATGAAACTACTACAGTGTGGCTAGTTCATAACACTGTTTGTCGAATGGTATTTATTACTT 60

gi|163247532|dbj|AB245013.2|:1036-1126 ATGAAACTACTACAGTGTGGCTAGTTCATAACACTGTTTGTCGAATGGTATTTATTACTT 60

gi|163247530|dbj|AB245011.2|:1036-1126 ATGAAACTACTACAGTGTGGCTAGTTCATAACACTGTTTGTCGAATGGTATTTATTACTT 60

gi|163247528|dbj|AB245009.2|:1036-1126 ATGAAACTACTACAGTGTGGCTAGTTCATAACACTGTTTGTCGAATGGTATTTATTACTT 60

gi|163247525|dbj|AB245006.2|:1036-1126 ATGAAACTACTACAGTGTGGCTAGTTCATAACACTGTTTGTCGAATGGTATTTATTACTT 60

gi|163247524|dbj|AB245005.2|:1036-1126 ATGAAACTACTACAGTGTGGCTAGTTCATAACACTGTTTGTCGAATGGTATTTATTACTT 60

gi|163247523|dbj|AB245004.2|:1036-1126 ATGAAACTACTACAGTGTGGCTAGTTCATAACACTGTTTGTCGAATGGTATTTATTACTT 60

gi|163247521|dbj|AB245002.2|:1036-1126 ATGAAACTACTACAGTGTGGCTAGTTCATAACACTGTTTGTCGAATGGTATTTATTACTT 60

gi|163247520|dbj|AB245001.2|:1036-1126 ATGAAACTACTACAGTGTGGCTAGTTCATAACACTGTTTGTCGAATGGTATTTATTACTT 60

gi|163247519|dbj|AB245000.2|:1036-1126 ATGAAACTACTACAGTGTGGCTAGTTCATAACACTGTTTGTCGAATGGTATTTATTACTT 60

gi|163247518|dbj|AB244999.2|:1036-1126 ATGAAACTACTACAGTGTGGCTAGTTCATAACACTGTTTGTCGAATGGTATTTATTACTT 60

gi|163247517|dbj|AB244998.2|:1036-1126 ATGAAACTACTACAGTGTGGCTAGTTCATAACACTGTTTGTCGAATGGTATTTATTACTT 60

gi|163247516|dbj|AB244997.2|:1036-1126 ATGAAACTACTACAGTGTGGCTAGTTCATAACACTGTTTGTCGAATGGTATTTATTACTT 60

gi|163247515|dbj|AB244996.2|:1036-1126 ATGAAACTACTACAGTGTGGCTAGTTCATAACACTGTTTGTCGAATGGTATTTATTACTT 60

gi|163247514|dbj|AB244995.2|:1036-1126 ATGAAACTACTACAGTGTGGCTAGTTCATAACACTGTTTGTCGAATGGTATTTATTACTT 60

gi|163247512|dbj|AB244993.2|:1036-1126 ATGAAACTACTACAGTGTGGCTAGTTCATAACACTGTTTGTCGAATGGTATTTATTACTT 60

gi|163247511|dbj|AB244992.2|:1036-1126 ATGAAACTACTACAGTGTGGCTAGTTCATAACACTGTTTGTCGAATGGTATTTATTACTT 60

gi|163247510|dbj|AB244991.2|:1036-1126 ATGAAACTACTACAGTGTGGCTAGTTCATAACACTGTTTGTCGAATGGTATTTATTACTT 60

gi|163247509|dbj|AB244990.2|:1036-1126 ATGAAACTACTACAGTGTGGCTAGTTCATAACACTGTTTGTCGAATGGTATTTATTACTT 60

gi|11991176|gb|AF304564.1|AF304564:1015-1105 ATGAAACTACTACAGTGTGGCTAGTTCATAACACTGTTTGTCGAATGGTATTTATTACTT 60

gi|11991174|gb|AF304562.1|AF304562:1015-1105 ATGAAACTACTACAGTGTGGCTAGTTCATAACACTGTTTGTCGAATGGTATTTATTACTT 60

gi|11991172|gb|AF304560.1|AF304560:1015-1105 ATGAAACTACTACAGTGTGGCTAGTTCATAACACTGTTTGTCGAATGGTATTTATTACTT 60

gi|5019551|emb|X86027.1|:2-92 ATGAAACTACTACAGTGTGGCTAGTTCATAACACTGTTTGTCGAATGGTATTTATTACTT 60

gi|927090674|gb|KT428385.1|:1071-1161 ATGAAACTACTACAGTGTGGCTAGTTTATAACACTGTTTGTCGAATGGTATTTATTACTT 60

gi|758937928|gb|KP150498.1|:2-92 ATGAAACTACTACAGTGTGGCTAGTTTATAACACTGTTTGTCGAATGGTATTTATTACTT 60

gi|758937922|gb|KP150492.1|:2-92 ATGAAACTACTACAGTGTGGCTAGTTTATAACACTGTTTGTCGAATGGTATTTATTACTT 60

gi|507147018|gb|KC998717.1|:154-244 ATGAAACTACTACAGTGTGGCTAGTTTATAACACTGTTTGTCGAATGGTATTTATTACTT 60

gi|507147017|gb|KC998716.1|:154-244 ATGAAACTACTACAGTGTGGCTAGTTTATAACACTGTTTGTCGAATGGTATTTATTACTT 60

gi|507147016|gb|KC998715.1|:154-244 ATGAAACTACTACAGTGTGGCTAGTTTATAACACTGTTTGTCGAATGGTATTTATTACTT 60

gi|163247541|dbj|AB245022.2|:1036-1126 ATGAAACTACTACAGTGTGGCTAGTTTATAACACTGTTTGTCGAATGGTATTTATTACTT 60

gi|163247531|dbj|AB245012.2|:1036-1126 ATGAAACTACTACAGTGTGGGTAGTTCATAACACTGTTTGTCGAATGGTATTTATTACTT 60

gi|163247529|dbj|AB245010.2|:1036-1126 ATGAAACTACTACAGTGTGGATAGTTCATAACACTGTTTGTCGAATGGTATTTATTACTT 60

gi|163247527|dbj|AB245008.2|:1036-1126 ATGAAACTACTACAGTGTGGCTAGTTTATAACACTGTTTGTCGAATGGTATTTATTACTT 60

gi|163247526|dbj|AB245007.2|:1036-1126 ATGAAACTACTACAGTGTGGCTAGTTTATAACACTGTTTGTCGAATGGTATTTATTACTT 60

gi|11991177|gb|AF304565.1|AF304565:720-810 ATGAAACTACTACAGTGTGGCTAGTTTATAACACTGTTTGTCGAATGGTATTTATTACTT 60

gi|11991175|gb|AF304563.1|AF304563:811-901 ATGAAACTACTACAGTGTGGCTAGTTTATAACACTGTTTGTCGAATGGTATTTATTACTT 60

gi|11991173|gb|AF304561.1|AF304561:607-697 ATGAAACTACTACAGTGTGGCTAGTTTATAACACTGTTTGTCGAATGGTATTTATTACTT 60

gi|11991178|gb|AF304566.1|AF304566:1015-1105 ATGAAACTACTACTGTGTGGCTAGTTTATAACACTGTTTGTCGAATGGTATTTATTACTT 60

Forward primer *O. ostertagi* ATGAAACTACTACAGTGTGGCTAGTTCA

Probe *O. ostertagi* GTCGAATGGTATTTATTACT

************* ****** ***** *********************************

gi|163247540|dbj|AB245021.2|:1036-1126 TATTGTGATAATTCCCATTCCAGTTCAAGAA 91

gi|507147019|gb|KC998718.1|:154-244 TATTGTGATAATTCCCATTCCAGTTCAAGAA 91

gi|357429009|dbj|AB682689.1|:914-1004 TATTGTGATAATTCCCATTCCAGTTCAAGAA 91

gi|357429008|dbj|AB682688.1|:914-1004 TATTGTGATAATTCCCATTCCAGTTCAAGAA 91

gi|163247543|dbj|AB245024.2|:1036-1126 TATTGTGATAATTCCCATTCCAGTTCAAGAA 91

gi|163247542|dbj|AB245023.2|:1036-1126 TATTGTGATAATTCCCATTCCAGTTCAAGAA 91

gi|163247539|dbj|AB245020.2|:1036-1126 TATTGTGATAATTCCCATTCCAGTTCAAGAA 91

gi|163247537|dbj|AB245018.2|:1036-1126 TATTGTGATAATTCCCATTCCAGTTCAAGAA 91

gi|163247536|dbj|AB245017.2|:1036-1126 TATTGTGATAATTCCCATTCCAGTTCAAGAA 91

gi|163247535|dbj|AB245016.2|:1036-1126 TATTGTGATAATTCCCATTCCAGTTCAAGAA 91

gi|163247534|dbj|AB245015.2|:1036-1126 TATTGTGATAATTCCCATTCCAGTTCAAGAA 91

gi|163247533|dbj|AB245014.2|:1036-1126 TATTGTGATAATTCCCATTCCAGTTCAAGAA 91

gi|163247532|dbj|AB245013.2|:1036-1126 TATTGTGATAATTCCCATTCCAGTTCAAGAA 91

gi|163247530|dbj|AB245011.2|:1036-1126 TATTGTGATAATTCCCATTCCAGTTCAAGAA 91

gi|163247528|dbj|AB245009.2|:1036-1126 TATTGTGATAATTCCCATTCCAGTTCAAGAA 91

gi|163247525|dbj|AB245006.2|:1036-1126 TATTGTGATAATTCCCATTCCAGTTCAAGAA 91

gi|163247524|dbj|AB245005.2|:1036-1126 TATTGTGATAATTCCCATTCCAGTTCAAGAA 91

gi|163247523|dbj|AB245004.2|:1036-1126 TATTGTGATAATTCCCATTCCAGTTCAAGAA 91

gi|163247521|dbj|AB245002.2|:1036-1126 TATTGTGATAATTCCCATTCCAGTTCAAGAA 91

gi|163247520|dbj|AB245001.2|:1036-1126 TATTGTGATAATTCCCATTCCAGTTCAAGAA 91

gi|163247519|dbj|AB245000.2|:1036-1126 TATTGTGATAATTCCCATTCCAGTTCAAGAA 91

gi|163247518|dbj|AB244999.2|:1036-1126 TATTGTGATAATTCCCATTCCAGTTCAAGAA 91

gi|163247517|dbj|AB244998.2|:1036-1126 TATTGTGATAATTCCCATTCCAGTTCAAGAA 91

gi|163247516|dbj|AB244997.2|:1036-1126 TATTGTGATAATTCCCATTCCAGTTCAAGAA 91

gi|163247515|dbj|AB244996.2|:1036-1126 TATTGTGATAATTCCCATTCCAGTTCAAGAA 91

gi|163247514|dbj|AB244995.2|:1036-1126 TATTGTGATAATTCCCATTCCAGTTCAAGAA 91

gi|163247512|dbj|AB244993.2|:1036-1126 TATTGTGATAATTCCCATTCCAGTTCAAGAA 91

gi|163247511|dbj|AB244992.2|:1036-1126 TATTGTGATAATTCCCATTCCAGTTCAAGAA 91

gi|163247510|dbj|AB244991.2|:1036-1126 TATTGTGATAATTCCCATTCCAGTTCAAGAA 91

gi|163247509|dbj|AB244990.2|:1036-1126 TATTGTGATAATTCCCATTCCAGTTCAAGAA 91

gi|11991176|gb|AF304564.1|AF304564:1015-1105 TATTGTGATAATTCCCATTCCAGTTCAAGAA 91

gi|11991174|gb|AF304562.1|AF304562:1015-1105 TATTGTGATAATTCCCATTCCAGTTCAAGAA 91

gi|11991172|gb|AF304560.1|AF304560:1015-1105 TATTGTGATAATTCCCATTCCAGTTCAAGAA 91

gi|5019551|emb|X86027.1|:2-92 TATTGTGATAATTCCCATTCCAGTTCAAGAA 91

gi|927090674|gb|KT428385.1|:1071-1161 TATTGTGATAATTCCCATTCCAGTTCAAGAA 91

gi|758937928|gb|KP150498.1|:2-92 TATTGTGATAATTCCCATTCCAGTTCAAGAA 91

gi|758937922|gb|KP150492.1|:2-92 TATTGTGATAATTCCCATTCCAGTTCAAGAA 91

gi|507147018|gb|KC998717.1|:154-244 TATTGTGATAATTCCCATTCCAGTTCAAGAA 91

gi|507147017|gb|KC998716.1|:154-244 TATTGTGATAATTCCCATTCCAGTTCAAGAA 91

gi|507147016|gb|KC998715.1|:154-244 TATTGTGATAATTCCCATTCCAGTTCAAGAA 91

gi|163247541|dbj|AB245022.2|:1036-1126 TATTGTGATAATTCCCATTCCAGTTCAAGAA 91

gi|163247531|dbj|AB245012.2|:1036-1126 TATTGTGATAATTCCCATTCCAGTTCAAGAA 91

gi|163247529|dbj|AB245010.2|:1036-1126 TATTGTGATAATTCCCATTCCAGTTCAAGAA 91

gi|163247527|dbj|AB245008.2|:1036-1126 TATTGTGATAATTCCCATTCCAGTTCAAGAA 91

gi|163247526|dbj|AB245007.2|:1036-1126 TATTGTGATAATTCCCATTCCAGTTCAAGAA 91

gi|11991177|gb|AF304565.1|AF304565:720-810 TATTGTGATAATTCCCATTCCAGTTCAAGAA 91

gi|11991175|gb|AF304563.1|AF304563:811-901 TATTGTGATAATTCCCATTCCAGTTCAAGAA 91

gi|11991173|gb|AF304561.1|AF304561:607-697 TATTGTGATAATTCCCATTCCAGTTCAAGAA 91

gi|11991178|gb|AF304566.1|AF304566:1015-1105 TATTGTGATAATTCCCATTCCAGTTCAAGAA 91

Reverse primer *O. ostertagi* TGATAATTCCCATTCCAGTTCAAGAA

*******************************
